# A novel approach to study the structure-property relationships and applications in living systems of modular Cu^2+^ fluorescent probes

**DOI:** 10.1038/srep28972

**Published:** 2016-08-03

**Authors:** Mengyao She, Zheng Yang, Likai Hao, Zhaohui Wang, Tianyou Luo, Martin Obst, Ping Liu, Yehua Shen, Shengyong Zhang, Jianli Li

**Affiliations:** 1 Ministry of Education Key Laboratory of Synthetic and Natural Functional Molecule Chemistry, College of Chemistry & Materials Science, Northwest University, Xi’an, Shaanxi 710127, P. R. China; 2 College of Chemistry and Chemical Engineering, Xi’an University of Science and Technology, Xi’an, Shaanxi 710054, P. R. China; 3 Center for Applied Geoscience, Institute for Geoscience, Eberhard Karls University Tuebingen, Hoelderlinstr. 12, Tuebingen 72074, Germany; 4 Bayreuth Center for Ecology and Environmental Research (BayCEER), University of Bayreuth, Dr.-Hans-Frisch-Str. 1-3, Bayreuth 95448, Germany

## Abstract

A series of Cu^2+^ probe which contains 9 probes have been synthesized and established. All the probes were synthesized using Rhodamine B as the fluorophore, conjugated to various differently substituted cinnamyl aldehyde with C=N Schiff base structural motif as their core moiety. The structure-property relationships of these probes have been investigated. The change of optical properties, caused by different electronic effect and steric effect of the recognition group, has been analyzed systematically. DFT calculation simulation of the Ring-Close and Ring-Open form of all the probes have been employed to illuminate, summarize and confirm these correlations between optical properties and molecular structures. In addition, biological experiment demonstrated that all the probes have a high potential for both sensitive and selective detection, mapping of adsorbed Cu^2+^ both *in vivo* and environmental microbial systems. This approach provides a significant strategy for studying structure-property relationships and guiding the synthesis of probes with various optical properties.

Selective recognition and detection of cations by receptors have attracted a significant amount of attention in chemistry, biology, and environmental science^1^,^2^,^3^,^4^. Among various detection receptors, fluorescent probe, based ion-induced changes in absorption or fluorescence spectra, appears to be particularly attractive due to their simplicity, high sensitivity, excellent selectivity, and instantaneous response^5^,^6^,^7^. However, properties of a fluorescent probe depend strongly on the binding properties of the target cation and the recognizing site in probe molecular^8^,^9^. Therefore, the binding properties and the geometrical configuration of the coordination sites in the corresponding probes are the most important parameters to consider during design and synthesis fluorescent probes. A commonly strategy is to attach a ligand in fluorophore to form the target recognizing site in the design of fluorescent probes^10^,^11^,^12^. High sensitivity and selectivity are fundamental goals and core design principles for an excellent probe. Though a large amount of researches had reported in the past decades, these researches did not mention electronic and steric effects that can regulate the performance in the recognition process^13^,^14^,^15^,^16^.

Copper, as the third most abundant essential trace element in human body, plays a crucial role for a broad range of biological processes^17^,^18^,^19^,^20^. However, copper excess cause toxicity to most living organisms, and copper release can eventually lead to serious environmental contamination^21^,^22^,^23^. Therefore, a convenient and rapid method for the detection of copper became increasingly important in biological and environmental concerns. Though chelating of Cu^2+^ with chemo sensors is well known to induce intrinsic fluorescence quenching due to the paramagnetic nature of Cu^2+ ^24, hundreds of fluorescent probes have been developed for varying purposes within the last few decades^25^,^26^,^27^. Systematic studies of these effects thus have expected to get great significance for the further development of Cu^2+^ fluorescent probes. Evermore, selection of an adequate probe for CLSM studies in bio-, geo-, and environmental sciences became more and more challenging^28^.

In order to investigate the effect of structure on performance for a probe, we synthesized and established a series of Cu^2+^ probe which contains 9 probes**1a-1i** based on our previous work (Fig. 1)^29^. Each of them is synthesized using rhodamine B as the fluorophore, conjugated to various differently substituted cinnamyl aldehyde with C=N Schiff base structural motif as its core segment. These probes show superior stable optical properties for the detection of Cu^2+^ which are not affect by fluorescence quenching. All these probes are synthesized easily under chemically mild conditions with a high yield (>80%) through a two-step reaction via Rhodamine B with substituted cinnamyl aldehyde. To our surprise, all probes showed different absorption and fluorescence responsive intensity to Cu^2+^.

In this work, we focused on the structure-property relationships of this probe model, investigated the change of optical properties caused by different electronic effect and steric effect of the recognition group, and also employed DFT calculation simulate the Ring-Close and Ring-Open form of all the probes (Fig. 2). Comprehensive analyses on the regulatory effects of substituent group about this probe model have been elaborated using frontier molecular orbital theory, NBO charge population and Fukui functional analysis.

## Results and Discussion

### UV-vis properties

Binding affinities of each probe toward varies metal ions, namely the chloride salts of Li^+^, Na^+^, K^+^, Ba^2+^, Ca^2+^, Cd^2+^, Mg^2+^, Co^2+^, Mn^2+^, Zn^2+^, Pb^2+^, Ni^2+^, Fe^2+^, Hg^2+^, Fe^3+^, Al^3+^, Cr^3+^, Cu^2+^ and the nitrate salt of Ag^+^ were evaluated by UV-vis spectroscopy. Upon addition of the respective metal ions, the absorption spectrum of the individual probes changed in different manners as shown in Figure S9. However, all tested metal ions but Cu^2+^ showed only minor spectral or color changes. Similar responses were observed when Cu^2+^ ions were added to the probe solutions in the presence of other metal ions. The absorption spectrum of probe 1a-1i exhibited a broad band at 556 nm at room temperature in the PBS buffer solution (pH = 7.4, 50% (v/v) ethanol). Since the closed form of rhodamine did not show an intense absorption band in the visible region^30^, this result suggested that probe Cu^2+^ complexes were not present in the form of closed spirolactone, but in a opened quinoid configuration at neutral pH. Furthermore, the absorption response all individual probes toward Cu^2+^ showed linear relationships in the range 0–50.0 μM of Cu^2+^ (all the graphs are showed in Supplementary Information).

**Figure 3:**
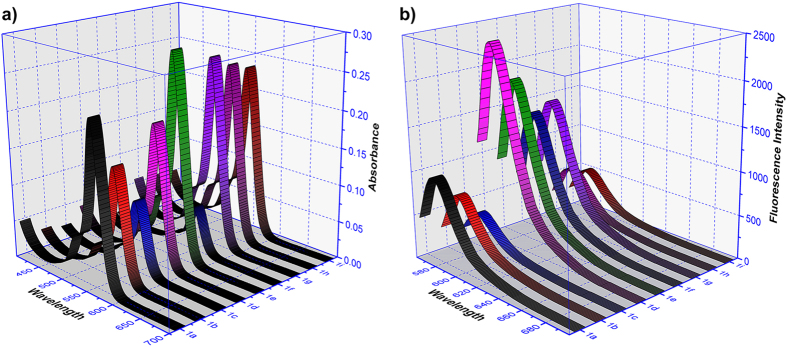
Absorbance and fluorescence intensities of probes **1a-1i** (10 μM) + Cu^2+^ (50 μM) in ethanol-PBS (5/5, v/v, pH 7.4), (**a**) Absorbance: maximum absorption at 556 nm; (**b**) fluorescence intensities: λ_ex_ = 550 nm, λ_em_ = 576 nm.

More importantly, the UV absorbance of these probes were shown to be adjusted by the different substituent groups. As shown in (Fig. 3a), the comparison of probe **1a**, **1b**, **1c** indicates that with a volume increases of **R**_**1**_, the strength of UV absorbance decreases; the reasons for this phenomenon may be caused by the increase of steric hindrance effects that obstruct the combination of probe with Cu^2+^. In other words, **R**_**1**_ plays a negative, volume-dependent role in the detection of Cu^2+^. When other heavy elements used as substituent at position **R**_**1**_ instead, the UV absorption spectrum in presence of Cu^2+^ showed a strong absorption band. In this study, we used Br as a substituent for instance. Moreover, we recognized visually that electronegative groups in the specific recognition unit of probe had a positive effect on the UV absorbance whereas electron-donating groups have a negative effect (probe **1e**: **R**_**1**_=NO_2_, probe **1f**: **R**_**1**_=OCH_3_). In addition, the absorbance of the probe + Cu^2+^ complexes were reduced gradually (**1g** > **1h** > **1i**), when methyl was introduced into different positions of benzene ring, whereas in ortho-, meta-, para-position it had scarcely any effect on the UV absorbance response in this series of probes.

### Fluorescence properties

The fluorescence properties of the probes and their respective responses to Cu^2+^ was evaluated in ethanol-PBS (5/5, v/v, pH 7.4) solution. The remarkable orange fluorescence emission of our probes increase that is induced by Cu^2+^ can interpreted as the opening of the spiral ring at appropriate pH; the emission band of this probe series appeared at 576 nm. The changes of **1a-1i** (10 μM) upon addition of Cu^2+^ (0–5.0 equiv.) have been studied (Figure S9). Fluorescence titration spectra of **1a-1i** with Cu^2+^ from 0 μM to 50 μM showed that these spectroscopic responses increased with increasing concentration of Cu^2+^ for each probe in this series. We also performed competition experiments with metal ions other than Cu^2+^ under the same conditions as for the absorbance study. The result revealed that this series of probes has significant anti-jamming capabilities in the presence of 5.0 equiv. of other ions (Figure S5). Furthermore, concentrations of Cu^2+^ and fluorescence intensities have a good linear relationship as shown in Figure S6. The coordination also shown to be a reversible process. When excess ethylenediamine was added to the colored solution of the complex, the fluorescence disappeared and the pink solution turned back to colorless owing to the de-coordination of Cu^2+^. Thus, **1a-1i** can be classified as a conditional reversible probe for Cu^2+^.

As show in (Fig. 3b), fluorescence intensity of probe **1a**-**1c** was reduced by increasing steric hindrance of substituent group **R**_**1**_. In addition, heavy elements substituting for **R**_**1**_ (e.g. Br) enhanced the fluorescence response significantly. For comparison, we have synthetized a congeneric probe **1j** which is substituted by Cl at the same position of Br and exhibited a lower fluorescence intensity than that of **1d**. Similarly, we introduced -NO_2_ and -OCH_3_ substituents to the recognition unit of the molecule to analyze the relationship between fluorescence intensity and electronic effects. The result suggested that electron-drawing groups promote fluorescence enhancement to a higher extent as compared to electron-donating groups. Substitutions in the ortho-position of the benzene ring increased the fluorescence intensity, whereas this effect was not observed when the substitution was inserted in meta- or para-position.

### Theoretical calculation analysis

To better understand the structure-property relationships of these 9 probes, the mode of ring-close and ring-open were studied by DFT^31^ calculations, all the calculations were performed with B3LYP functional with a combination of basis of double-ζ quality consisting of 6-31G^**^ for C, H elements and 6-31+G* for N, O, Br elements on Gaussian 09 Program^32^. (Full citations are given in supporting information). The optimized structures were confirmed to be local minimums due to the non-existence of imaginary frequency. The environmental effect was included via PCM model with ethanol as the solvent molecule.

As show in Table 1, all the Ring-Open forms possesses higher energy than that of Ring-Close form over 12 Kcal/mol. These results indicated that these probes prefer to present in a lactam form without environmental disturbance. Especially, probe **1a-1c** show gradualness energy increase, which may be attributed to the block transition process by the growing steric hindrance. Extraordinary for probe **1d**, the exist of heavy atom Br could promote the equalization of electronic cloud distribution and reduce energy accordingly, and the energy required for the formation of Ring-Open structure is less than any other probe, which indicated that probe **1d** may show a better optical property because of its easy changed structure. The molecule energy seems to be insensitive to the Push-pull electronic effect and spatial effect on the benzene ring of recognition group.

**Figure 4:**
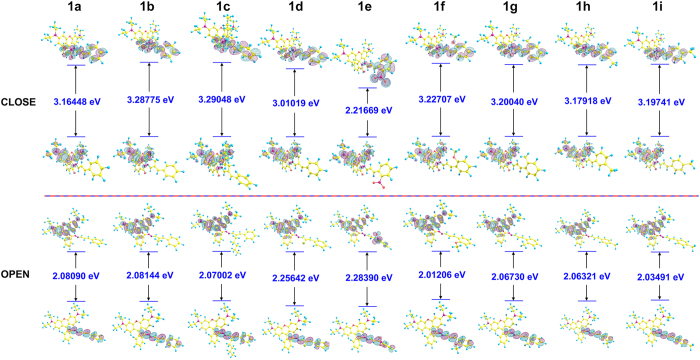
The HOMO-LUMO Gaps of probe 1a-1i.

The HOMO-LUMO orbital energies and the spatial distributions for these probes were determined; the spatial distributions HOMO and LUMO were displayed in Fig. 4. In the Ring-Close form, all HOMOs were distributed to xanthene moiety and LUMOs were located on the recognition part. The distribution was exactly the opposite in the Ring-Open form. It’s obvious that all the Ring-Open structures have higher HOMO energy and lower LUMO energy except probe **1e**. The LUMO energy of probe **1e** showed an abnormal reduction from other probes, which may due to the evenly distributed electron density caused by the strong electrophilic effect of NO_2_.

**Figure 5:**
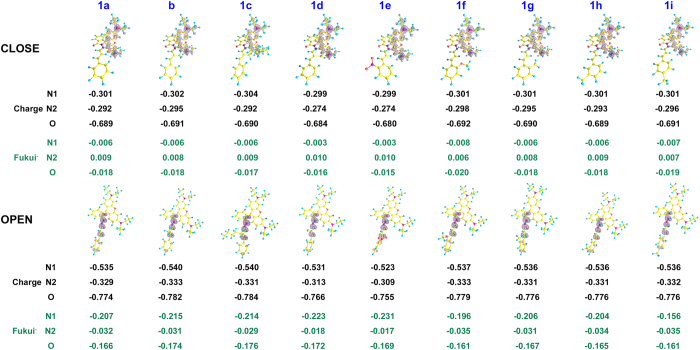
NBO charge, 3D representation of the Fukui function *f*^−^_(r)_ of the iso-value of 0.003 a.u. (positive in red color and negative in green color) and the D-value condensed Fukui function *f*^*−*^_(r)_ of O, N_1_ and N_2_.

The results of NBO analysis^33^ demonstrate the polarization of Charge distribution (Fig. 5), the electron was flowing from xanthene moiety to the recognized moiety during the process of transform, the charge is which was consistent with reported literature. The larger density of electron cloud represents the higher electron donating ability.

On the other hand, *f*^−^_(r)_ has been successfully used to describe the reactivity concerning electrophilic attack^34^, CuCl_2_ attack in this model of probe. The condensed Fukui function of individual atom is obtained from NBO analysis. As show in Fig. 5 the 3D representation of the *f*^−^_(r)_ clearly demonstrates that the region around carbonyl O atom and hydrazide N atom possesses higher reactivity than other parts of the Ring-Open form and there is no obvious electrophilic site in the recognized moiety of Ring-Close form. These data illustrate that all the probes have the tendency to be opened rather than closed under the entrainment and coordination of Cu^2+^.

### CLSM bioimaging applications

**Figure 6:**
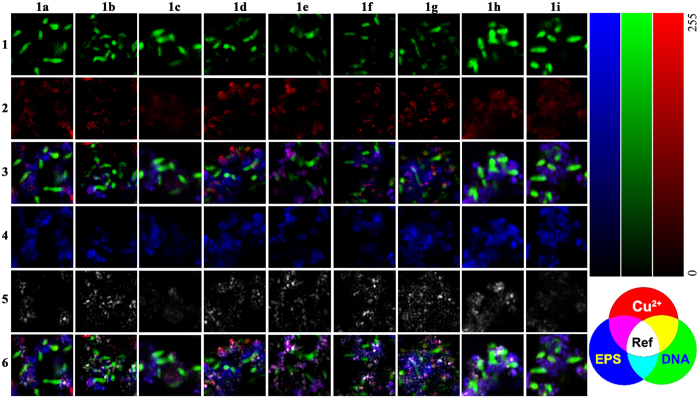
Single cell scale maps showing the sorption of Cu^2+^ to cell-EPS-mineral aggregates formed by Fe(II)-oxidizing Rhodobacter sp. strain SW2 incubated with 50 μM CuCl_2_ for 1 h at 25 °C. The aggregate was simultaneously incubated with 50 μM of probes **1a**-**1i** (2) and 20% C_2_H_5_OH, fluorescent nucleic acid stain (1) and lectin conjugate (4) for 1 h at 25 °C, minerals of biogenic origin were visualized employing their reflection signal (5) (λ_ex_ = 488 nm, 561 nm, 635 nm). The overlay image of (1) (2) and (4) is shown in (3); the overlay image of all four signals is shown in (6). Brighter colour indicates a higher metal concentration.

These probes were selected for Cu^2+^-localization studies in microbial cell-extracellular polymeric substances (EPS)-mineral aggregates. The photosynthetic Fe(II)-oxidizing Rhodobacter sp. strain SW2 was selected as an model organism because SW2 is known to form such aggregates^35^ and for being highly resistant to heavy metals during its Fe(II) oxidization^36^. It was incubated under permanent illumination in fresh water mineral medium for 1 week. The resulting suspension of aggregates that consisted of microbial cells, extracellular polymers and Fe(III)-minerals was equilibrated with 50 μM of CuCl_2_ for 30 min. Probe **1a-1i** (50 μM), SYTO 9 green fluorescent nucleic acid stain (1:500) and polysaccharide-specific Wheat germ agglutinin, ALEXA FLUOR 633 conjugate (1:200)^28^,^29^ were applied to visualize the distribution of Cu^2+^, bacterial cells and EPS respectively. The CLSM images show that probe **1a-1i** was able to detect Cu^2+^ in the aqueous microbial cell-EPS-mineral aggregates. The results furthermore indicate that Cu^2+^ is collocated with the bacterial cells and with the free, cloud-like EPS, suggesting that the organic surfaces provide binding sites for Cu^2+^, even though the detailed spatial distribution of Cu^2+^ on subcellular level and microbial sorption mechanism still remain unclear and need to be analyzed by higher resolution analytical microscopy approaches (Fig. 6). In summary, probe **1a**-**1i** can be used to label Cu^2+^-biosorption in microbial cell-EPS-mineral aggregates and biofilms. Since their fluorescence spectra are compatible for the combination with commercial available DNA- and polysaccharide-specific fluorescent dyes, they further provide the possibility to explore the correlation between Cu^2+^ and organic components in this system on the single cell level under natural and hydrated conditions. Our approach also provides a successful case wherein it is shown how to design, synthesize and improve other metal fluorescent probes with high sensitivity and selectivity. Finally, our attempt also revealed that the library-based strategy^37^ for imaging-based fluorescence screening is a powerful tool to select the most appropriate fluorescent metal probes for CLSM studies.

### Living cells bioimaging applications

**Figure 7:**
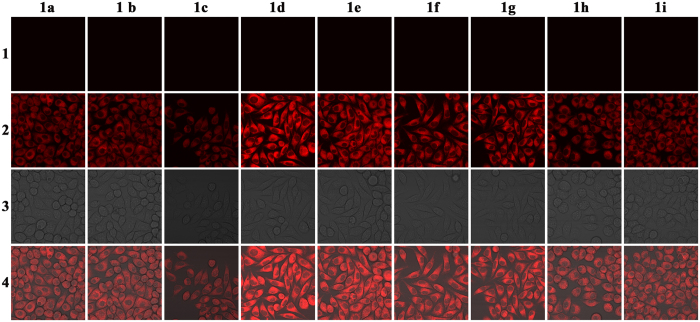
Fluorescent images of L929 cells incubated with 10 μM probes for 30 min (1) and then further incubated with 50 μM Cu^2+^ for 30 min (2). (3) Bright field image of cells. The overlay image of (2) and (3) is shown in (4) (λ_ex_ = 543 nm).

Next, we proceeded to investigate the utility of probe **1a-1i** in intracellular imaging with L929 to examine whether it can work in biological systems, the distribution of the probe within the cells was observed by laser scanning confocal microscopy following excitation at 543 nm. As show in Fig. 7, all the probes can permeate cellular membrane due to the molecule as well hydrophobicity, following addition of exogenous Cu^2+^, show relatively obvious intracellular fluorescence with excitation. Bright-field transmission measurements after the probe incubation confirm that the cells are viable. The overlay of fluorescence and bright-field images revealed that the fluorescence signals were localized in the perinuclear region of the cytosol, indicating the subcellular distribution of Cu^2+^ which was internalized into the living cells from the growth medium. We anticipate that these probes will be of great benefit for biomedical researchers investigating the effects of Cu^2+^ in biological systems.

## Methods

### Materials and Methods

All the reagent grade chemicals consumed in this work were procured from commercial sources and used as received. Cu^2+^ stock solution is prepared to be 200 μmol/L with ethanol using CuCl_2_ as the source, and probe **1a**-**1i** stock solution (50 ml) were prepared to be 200 μmol/L with ethanol and solubilized by little acetone (0.1 ml). The solutions of metal ions were prepared in ethanol for the selectivity and competitive study with chloride salts of Li^+^, Na^+^, K^+^, Ba^2+^, Ca^2+^, Cd^2+^, Mg^2+^, Co^2+^, Mn^2+^, Zn^2+^, Pb^2+^, Ni^2+^, Fe^2+^, Hg^2+^, Fe^3+^, Al^3+^, Cr^3+^, Cu^2+^ and the nitrate salt of Ag^+^.

To a 10 mL volumetric tube, different concentration of Cu^2+^, 5.0 mL PBS and 0.50 mL of 200 μM probes were added. The mixtures were diluted to 10 mL with ethanol. Then, 3.0 mL of each solution were transferred to a 1 cm cuvette. The absorbance was recorded at 556 nm and the fluorescence intensity was recorded at 576 nm. The excitation and emission wavelength bandpasses were both set as 5.0 nm and the excitation wavelength was set at 550 nm.

A Beijing TAIKE XT-4 microscopy melting point apparatus was used to measure the melting points of the compounds. Mass spectrometry was performed on a BRUKER micrOTOF-Q II ESI-Q-TOF LC/MS/MS Spectroscopy. NMR spectra were recorded on a VARIAN INOVA-400 spectrometer, using TMS (TMS trimethylsilyl) as an internal standard. The absorbance spectra were recorded on a SHIMADZU UV-1700 spectrophotometer. A HITACHI F-4500 FL spectrophotometer employed to record the fluorescence spectra. A VARIO EL III analyzer was used for performing the elemental analyses. Samples for mapping Cu^2+^ sorption to cell-mineral aggregates of Fe(II)-oxidizing bacteria were prepared under anoxic conditions in a glovebox under N_2_ atmosphere. The samples were analyzed using an upright confocal laser scanning microscope (LEICA SPE, Wetzlar, Germany), equipped with 4 lasers (405 nm, 488 nm, 561 nm, 635 nm) and a 63x water immersion objective (ACS APO 63x, NA = 1.15).

## Conclusions

In summary, we have synthesized and evaluated a series of Cu^2+^ probe which contains 9 probes, all the probes are based on the core segment of C=N Schiff base structural motif. This study revealed the relation between the structure and the optical properties of nine probes focusing on the influence of various substituents and provided a significant strategy for studying structure-property relationships which were illuminated, summarized and confirmed by DFT calculation simulation on the Ring-Close and Ring-Open form of this modular Schiff base Cu^2+^ fluorescent probes. We expect that these results of our research would help to guide the synthesis of high performance metal fluorescence probes with various properties. On the other hand, all the probes could permeate cellular membrane to investigating the effects of Cu^2+^ in living cells. Furthermore, CLSM experiments suggested that all the probes would be a powerful tool for sensitive and selective detection and mapping of Cu^2+^ adsorbed in environmental microbial systems.

## Additional Information

**How to cite this article**: She, M. *et al*. A novel approach to study the structure-property relationships and applications in living systems of modular Cu^2+^ fluorescent probes. *Sci. Rep.* **6**, 28972; doi: 10.1038/srep28972 (2016).

## Supplementary Material

Supplementary Information

## Figures and Tables

**Figure 1:**
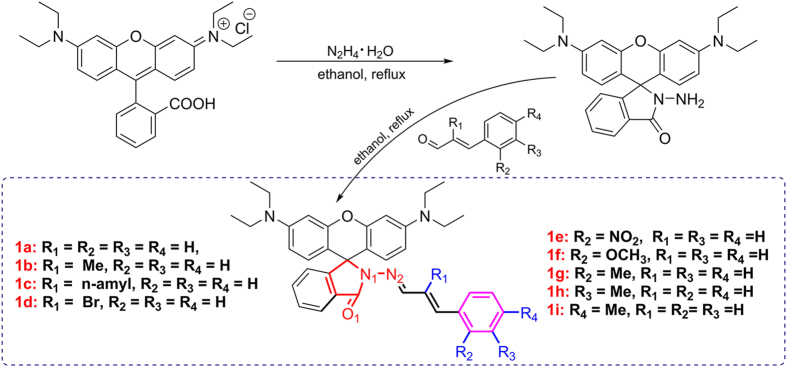
Synthesis route and molecular structure of probes 1a-1i.

**Figure 2:**
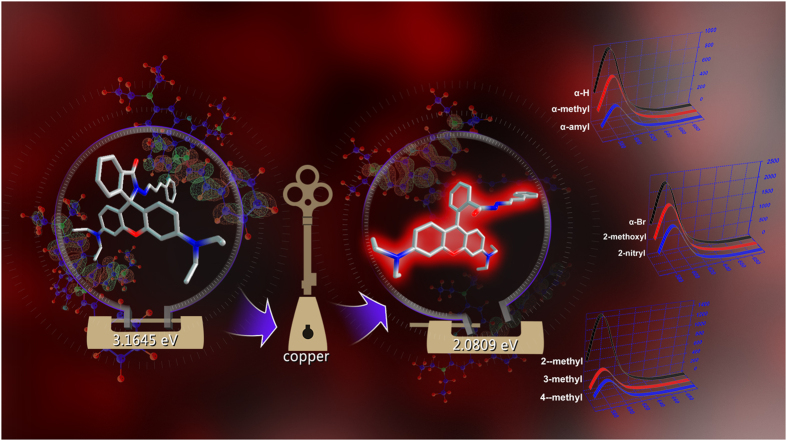
Schematic of the strategy for studying structure-property relationships of 9 modular Cu^2+^ fluorescent probes.

**Table 1:** The calculated energy(E) of all structures.

No.	Ring-close form (C)	Ring-open form (O)	ΔE (Kcal/mol)
	Total Energy (a.u.)	Total Energy (a.u.)	
1	−1802.3177	−1802.2962	**13.5098**
2	−1841.6321	−1841.6095	**14.2351**
3	−1998.8926	−1998.8673	**15.8898**
4	−4373.1169	−4373.0969	**12.5093**
5	−2006.8251	−2006.8018	**14.6411**
6	−1916.8454	−1916.8230	**14.0357**
7	−1841.6329	−1841.6110	**13.7240**
8	−1841.6377	−1841.6160	**13.6003**
9	−1841.6383	−1841.6162	**13.8344**
