# Supplementary material for: A novel approach to study the structure-property relationships and applications in living systems of modular Cu2+ fluorescent probes
Source: Sci Rep. 2016 Aug 3;6:28972. doi: 10.1038/srep28972 (PMC4971463; doi:10.1038/srep28972)
Supplement: Supplementary Information [file srep28972-s1.pdf]

Supplementary Information for

A novel approach to study the structure-property relationships and applications in living systems of modular Cu^{2+} fluorescent probes

Mengyao She[1,+], Zheng Yang[1,3,+], Likai Hao[2], Zhaohui Wang[1], Tianyou Luo[1], Martin Obst[2], Ping Liu[1],Yehua Shen[1], Shengyong Zhang[1], and Jianli Li[1,*]

[1]Ministry of Education Key Laboratory of Synthetic and Natural Functional Molecule Chemistry, College of Chemistry & Materials Science, Northwest University, Xi'an, Shaanxi 710127, P. R. China.

[2]Center for Applied Geoscience, Institute for Geoscience, Eberhard Karls University Tuebingen, Hoelderlinstr. 12, Tuebingen 72074, Germany.

[3]College of Chemistry and Chemical Engineering, Xi'an University of Science and Technology, Xi'an, Shaanxi 710054, P. R. China.

*Correspondence to: Prof. Jianli Li. Tel.: +86 029 81535026; Fax: +86 029 88308396. E-mail: lijianli@nwu.edu.cn.

Content

1. Synthesis of probe **1a-1i** 2
2. Spectroscopic properties of **1a-1i** 6
3. Equations used for the calculation of association constant about **1a-1i** 19
4. Equations used for the calculation of fluorescence quantum yield 20
5. Calculation of the detection limit of probe **1a-1i** 20
6. IR, ^1H NMR, ^{13}C NMR and MS spectra of all the compounds 21
7. Data, spectrum and spectroscopic properties of **1j** for comparison 39
8. Full citation information of Gaussian 09 program 42

1. Synthesis of probe **1a-1i**

Synthesis of N-(rhodamine-B)lactam-hydrazine

Rhodamine B (4.78 g, 0.01 mol) was dissolved in 50 mL ethanol solution. Then, a solution of hydrazine hydrate (8.0 mL) was added dropwised over 30 min. The reaction mixture was refluxed for 4 h until the fluorescence of the solution disappeared. Then, the solvent was evaporated under reduced pressure resulting in a red oil, which was then recrystallized from ethanol/water to afford N-(rhodamine-B)lactam-hydrazine as a white crystal (7.36 g, yield: 80.7%), mp: 198-199 ℃. Anal. calcd. for $C_{28}H_{32}N_4O_2$: H, 7.06; C, 73.66; N, 12.27. Found: H, 7.08; C, 73.50; N, 12.29. ^{1}H NMR ($CDCl_3$, 400 MHz, TMS): δ (ppm) = 8.03-7.86 (m, 1H), 7.61-7.29 (m, 2H), 7.22-7.02 (m, 1H), 6.44 (dd, *J* = 17.2, 5.6 Hz, 4H), 6.29 (dd, *J* = 8.8, 2.4 Hz, 2H), 3.60 (d, *J* = 8.0 Hz, 2H), 3.34 (q, *J* = 7.0 Hz, 8H), 1.17 (t, *J* = 7.0 Hz, 12H). ^{13}C NMR ($CDCl_3$, 100 MHz, TMS): δ (ppm) = 165.4, 153.2, 150.9, 148.2, 131.8, 129.3, 127.4, 123.1, 122.3, 107.3, 103.9, 97.3, 65.2, 43.7, 11.9. IR (cm-1): 3432.8, 3395.6, 3080.3, 2969.4, 2925.7, 1691.6, 1612.9, 1515.2, 1307.3, 1267.5, 1222.5, 1118.5, 817.4, 785.9, 756.4, 694.4.

*Synthesis of probes **1a-1i***

N-(rhodamine-B)lactam-hydrazine (4.56 g, 0.01 mol) and the cinnamyl aldehyde derivatives (3.0 mL) were mixed in 50 mL ethanol and the reaction mixture was refluxed for 6 h. After cooling to the room temperature, the solvent was evaporated and the resulting liquid was purified by column chromatography on silica gel (eluent: $CHCl_3$:CH_3OH = 30:1).

1a: White powder, 5.28 g, yield: 92.6%. mp: 204-205 ℃. Anal. calc. for $C_{37}H_{38}N_4O_2$: H, 6.71; C, 77.87; N, 9.82. Found: H, 6.72; C, 77.78; N, 9.83. MS (ESI) m/z = 571.3608 $[M+H]^+$, calc. for $C_{37}H_{38}N_4O_2$=570.7232. ^{1}H NMR (400 MHz, $CDCl_3$, TMS): δ (ppm) = 8.14 (d, *J* = 9.1 Hz, 1H), 7.99 (dd, *J* = 6.4, 1.6 Hz, 1H), 7.53 - 7.38 (m, 2H), 7.35 (d, *J* = 7.2 Hz, 2H), 7.31 - 7.15 (m, 3H), 7.10 - 6.99 (m, 1H), 6.88 (dd, *J* = 16.0, 9.1 Hz, 1H), 6.57 (dd, *J* = 18.7, 12.5 Hz, 3H), 6.44 (d, *J* = 2.5 Hz, 2H), 6.26 (dd, *J* = 8.9, 2.5 Hz, 2H), 3.51-3.14 (m, 8H), 1.26 - 0.96 (m, 12H). ^{13}C NMR (100 MHz, $CDCl_3$, TMS): δ (ppm) = 165.3, 152.6, 148.9, 148.7, 138.8, 136.2, 133.4, 128.6, 128.1, 127.8, 127.3, 126.8, 123.5, 108.1, 105.5, 97.9, 65.6, 44.3, 12.6. IR (cm^{-1}): 3029.9, 2970.7, 2930.4, 1682.3, 1615.3, 1513.6, 1302.9, 1266.2, 1219.9, 1118.7, 824.7, 784.5, 753.4, 695.6.

1b: White powder, 5.34 g, yield: 91.4%. mp: 215-216 ℃. Anal. calcd. for $C_{38}H_{40}N_4O_2$: H, 6.89;

C, 78.05; N, 9.58. Found: H, 6.91; C, 77.99; N, 9.60. MS (ESI) m/z = 585.3217 $[M+H]^+$, calc. for $C_{37}H_{38}N_4O_2$=584.7498. ^1H NMR (400 MHz, $CDCl_3$): δ (ppm) = 8.56 (s, 1H), 8.08 - 7.94 (m, 1H), 7.63 - 7.41 (m, 2H), 7.38 - 7.30 (m, 4H), 7.27 - 7.20 (m, 1H), 7.19 - 7.07 (m, 1H), 6.53 (d, *J* = 8.8 Hz, 3H), 6.46 (d, *J* = 2.5 Hz, 2H), 6.28 (dd, *J* = 8.8, 2.5 Hz, 2H), 3.35 (q, *J* = 11.3 Hz, 8H), 2.01 (s, 3H), 1.18 (t, *J* = 7.0 Hz, 12H). ^{13}C NMR (100 MHz, $CDCl_3$, TMS): δ (ppm) = 164.6, 153.3, 153.0, 151.4, 148.9, 137.0, 136.4, 133.2, 129.9, 129.3, 128.2, 127.3, 123.9, 123.3, 107.9, 106.4, 97.8, 66.1, 44.4, 12.6. IR (cm^{-1}): 3022.9, 2969.4, 2928.1, 1689.5, 1613.6, 1515.3, 1303.9, 1264.5, 1220.6, 1118.1, 820.3, 786.1, 756.5, 695.7.

1c: White powder, 6.03 g, yield: 94.2%. mp: 172-173 ℃. Anal. calcd. for $C_{42}H_{48}N_4O_2$: H, 7.55; C, 78.71; N, 8.74. Found: H, 7.78; C, 78.62; N, 8.75. MS (ESI) m/z = 641.3864 $[M+H]^+$, calc. for $C_{37}H_{38}N_4O_2$=640.8561. ^1H NMR ($CDCl_3$, 400 MHz, TMS): δ (ppm) = 9.11 (s, 1H), 7.95 (m, 1H), 7.51 (m, 2H), 7.36-7.25 (m, 4H), 7.25-7.15 (m, 2H), 6.59 (s, 1H), 6.49 (d, *J* = 8.8 Hz, 2H), 6.42 (t, *J* = 6.6 Hz, 2H), 6.26 (dd, *J* = 8.8, 2.6 Hz, 2H), 3.33 (q, *J* = 7.0 Hz, 8H), 2.34 (t, *J* = 8.0 Hz, 2H), 1.28-1.21 (m, 6H), 1.18 (t, *J* = 6.0 Hz, 12H), 0.87 (t, *J* = 8.0 Hz, 3H). ^{13}C NMR ($CDCl_3$, 100 MHz, TMS): δ (ppm) = 164.1, 154.8, 153.8, 150.5, 148.7, 141.4, 137.0, 136.7, 132.9, 131.4, 123.0, 128.3, 127.2, 124.2, 123.1, 107.6, 106.9, 97.8, 66.8, 44.3, 32.3, 28.4, 26.0, 22.5, 14.3, 12.6. IR (cm^{-1}): 3021.5, 2967.2, 2927.5, 1688.2, 1615.2, 1514.5, 1307.1, 1265.5, 1220.8, 1119.7, 821.1, 785.5, 756.0, 697.8.

1d: Light yellow powder, 6.01 g, yield: 92.8%.mp: 212-213 ℃. Anal. Calc. for $C_{37}H_{37}BrN_4O_2$: H, 5.74; C, 68.41; N, 8.62. Found: H, 5.73; C, 68.45; N, 8.61. MS (ESI) m/z = 649.2299 $[M+H]^+$, Calc. for $C_{37}H_{37}BrN_4O_2$ = 648.2110. ^1H NMR (400 MHz, $CDCl_3$, TMS): δ (ppm) = 8.49 (s, 1H), 8.00 (d, *J* = 7.2 Hz, 1H), 7.73 (d, *J* = 7.2 Hz, 2H), 7.51 (t, *J* = 7.6 Hz, 2H), 7.33 (dd, *J* = 16.1, 8.5 Hz, 3H), 7.15 (d, *J* = 7.1 Hz, 1H), 7.06 (s, 1H), 6.50 (d, *J* = 8.8 Hz, 2H), 6.45 (s, 2H), 6.27 (dd, *J* = 8.8, 2.0 Hz, 2H), 3.34 (dd, *J* = 13.7, 6.8 Hz, 8H), 1.17 (t, *J* = 6.9 Hz, 12H). ^{13}C NMR (100 MHz, $CDCl_3$, TMS): δ (ppm) = 159.1, 147.8, 145.8, 143.4, 140.6, 131.1, 129.4, 127.9, 118.4, 117.9, 116.1, 102.4, 100.2, 92.3, 60.8, 38.8, 7.0. IR (cm^{-1}): 3074.46, 3033.60, 2967.65, 2873.87, 1721.73, 1615.33, 1546.64, 1516.31, 1466.78, 1424.94, 1364.74, 1304.35, 1218.20, 1119.60, 1017.28, 918.23, 895.65, 865.48, 845.92, 815.80, 788.17, 693.69.

1e: Yellow powder, 5.78 g, yield: 94.0%.mp: 221-223 ℃. Anal. Calc. for $C_{37}H_{37}N_5O_4$: H, 6.06; C, 72.17; N, 11.37. Found: H, 6.04; C, 72.16; N, 11.35. MS (ESI) m/z = 616.2937 $[M+H]^+$, Calc.

for $C_{37}H_{37}N_5O_4$ = 615.2846. ^{1}H NMR (400 MHz, $CDCl_3$, TMS): δ (ppm) = 8.22 (d, *J* = 9.0 Hz, 1H), 8.00 (d, *J* = 6.8 Hz, 1H), 7.86 (d, *J* = 7.9 Hz, 1H), 7.60 (d, *J* = 7.7 Hz, 1H), 7.55 - 7.39 (m, 3H), 7.34 (t, *J* = 7.7 Hz, 1H), 7.07 (d, *J* = 15.3 Hz, 2H), 6.87 (dd, *J* = 15.9, 9.0 Hz, 1H), 6.58 - 6.40 (m, 4H), 6.27 (d, *J* = 8.9 Hz, 2H), 3.33 (q, *J* = 7.0 Hz, 8H), 1.16 (t, *J* = 7.0 Hz, 12H). ^{13}C NMR (100 MHz, $CDCl_3$, TMS): δ (ppm) = 165.4, 152.7, 152.5, 149.0, 147.7, 147.4, 133.7, 133.0, 132.4, 132.0, 131.6, 127.8, 124.7, 123.7, 123.5, 108.0, 105.2, 98.0, 65.8, 44.3, 12.6. IR (cm^{-1}): 3076.88, 2971.12, 2931.54, 2360.98, 1685.24, 1614.37, 1513.65, 1464.34, 1397.37, 1375.92, 1338.18, 1304.21, 1266.32, 1216.54, 1116.53, 1019.33, 974.00, 830.45, 783.26, 743.18, 714.47, 684.10.

1f: Light yellow powder, 5.44 g, yield: 90.6%.mp: 214-215 ℃. Anal. Calc. for $C_{38}H_{40}N_4O_3$: H, 6.71; C, 75.97; N, 9.33. Found: H, 6.70; C, 76.02; N, 9.31. MS (ESI) m/z = 601.3189 $[M+H]^+$, Calc. for $C_{37}H_{38}N_4O_2$ = 600.3100. ^{1}H NMR (400 MHz, $CDCl_3$, TMS): δ (ppm) = 8.15 (d, *J* = 7.3 Hz, 1H), 7.99 (d, *J* = 6.8 Hz, 1H), 7.43 (dt, *J* = 14.7, 7.2 Hz, 3H), 7.22 (dd, *J* = 19.8, 11.9 Hz, 1H), 7.05 (d, *J* = 7.4 Hz, 1H), 7.00 - 6.74 (m, 4H), 6.56 (d, *J* = 8.8 Hz, 2H), 6.45 (d, *J* = 2.1 Hz, 2H), 6.26 (dd, *J* = 8.8, 2.2 Hz, 2H), 3.82 (s, 3H), 3.33 (dd, *J* = 14.0, 6.9 Hz, 8H), 1.17 (t, *J* = 7.0 Hz, 12H). ^{13}C NMR (100 MHz, $CDCl_3$, TMS): δ (ppm) = 160.3, 156.9, 152.6, 149.7, 148.9, 133.8, 133.3, 128.0, 127.2, 123.4, 120.7, 110.82, 108.1, 105.6, 98.0, 65.5, 55.4, 44.3, 12.6. IR (cm^{-1}): 3044.08, 2966.36, 2926.99, 1691.42, 1615.39, 1547.89, 1516.71, 1485.80, 1464.50, 1428.76, 1402.06, 1376.39, 1356.23, 1307.55, 1269.86, 1234.47, 1119.53, 1078.70, 986.10, 874.33, 823.04, 782.01, 754.49, 711.62, 698.64.

1g: Yellow powder, 5.35 g, yield: 91.6%.mp: 216-217 ℃. Anal. Calc. for $C_{38}H_{40}N_4O_2$: H, 6.89; C, 78.05; N, 9.58. Found: H, 6.88; C, 78.02; N, 9.59. MS (ESI) m/z = 585.3234 $[M+H]^+$, Calc. for $C_{38}H_{40}N_4O_2$ = 584.3151. ^{1}H NMR (400 MHz, $CDCl_3$, TMS): δ (ppm) = 8.39 (d, *J* = 8.5 Hz, 1H), 7.98 (d, *J* = 7.0 Hz, 1H), 7.44 (d, *J* = 5.6 Hz, 3H), 7.09 (dd, *J* = 17.5, 6.3 Hz, 4H), 6.80 (dt, *J* = 15.9, 12.3 Hz, 2H), 6.54 (d, *J* = 8.8 Hz, 2H), 6.44 (s, 2H), 6.34 - 6.18 (m, 2H), 3.33 (q, *J* = 6.9 Hz, 8H), 2.30 (s, 3H), 1.16 (t, *J* = 6.8 Hz, 12H). ^{13}C NMR (100 MHz, $CDCl_3$, TMS): δ (ppm) = 159.6, 147.2, 146.8, 144.5, 143.3, 130.8, 130.4, 129.3, 127.8, 122.8, 122.6, 122.4, 120.6, 119.9, 118.0, 117.8, 102.5, 100.2, 92.3, 60.4, 38.7, 11.2, 7.0. IR (cm^{-1}): 3050.26, 3016.20, 2969.99, 2925.94, 2887.37, 1687.52, 1615.42, 1546.72, 1514.95, 1479.57, 1463.22, 1424.08, 1398.74, 1375.34, 1354.17, 1306.01, 1266.92, 1215.95, 1116.73, 1018.08, 975.27, 874.92, 827.06, 783.86, 753.81, 714.25, 699.32.

1h: Yellow powder, 5.03 g, yield: 86.0%.mp: 123-124 ℃. Anal. Calc. for $C_{38}H_{40}N_4O_2$: H, 6.89; C, 78.05; N, 9.58. Found: H, 6.86; C, 78.09; N, 9.58. MS (ESI) m/z = 585.3235 $[M+H]^+$, Calc. for $C_{38}H_{40}N_4O_2$ = 584.3151. ^{1}H NMR (400 MHz, $CDCl_3$, TMS): δ (ppm) = 8.18 (d, *J* = 9.1 Hz, 1H), 8.05 - 7.91 (m, 1H), 7.43 (dd, *J* = 12.8, 6.3 Hz, 2H), 7.20 - 7.09 (m, 3H), 7.05 (d, *J* = 7.0 Hz, 2H), 6.85 (dd, *J* = 16.0, 9.1 Hz, 1H), 6.59 (s, 1H), 6.54 (d, *J* = 8.9 Hz, 2H), 6.43 (d, *J* = 2.4 Hz, 2H), 6.26 (dd, *J* = 8.9, 2.4 Hz, 2H), 3.33 (q, *J* = 7.0 Hz, 8H), 2.29 (s, 3H), 1.16 (t, *J* = 7.0 Hz, 12H). ^{13}C NMR (100 MHz, $CDCl_3$, TMS): δ (ppm) = 159.7, 147.1, 147.0, 143.4, 143.3, 133.4, 132.6, 130.5, 127.7, 123.8, 122.3, 121.6, 118.4, 118.0, 117.8, 102.5, 92.3, 60.0, 28.7, 15.7, 7.0. IR (cm-1): 3093.68, 3031.97, 2968.56, 2926.44, 1689.27, 1614.93, 1547.79, 1514.66, 1465.82, 1425.53, 1399.85, 1375.19, 1356.80, 1305.48, 1263.75, 1219.98, 1118.36, 976.42, 865.97, 820.49, 783.50, 756.87, 686.03.

1i: Yellow powder, 5.27 g, yield: 90.2%.mp: 125-126 ℃. Anal. Calc. for $C_{38}H_{40}N_4O_2$: H, 6.89; C, 78.05; N, 9.58. Found: H, 6.88; C, 78.08; N, 9.55. MS (ESI) m/z = 585.3236 $[M+H]^+$, Calc. for $C_{38}H_{40}N_4O_2$ = 584.3151. ^{1}H NMR (400 MHz, $CDCl_3$, TMS): δ (ppm) = 8.17 (d, *J* = 9.1 Hz, 1H), 7.99 (d, *J* = 6.9 Hz, 1H), 7.44 (dt, *J* = 13.1, 6.6 Hz, 3H), 7.18 - 6.99 (m, 3H), 6.97 - 6.68 (m, 2H), 6.62 - 6.49 (m, 2H), 6.44 (s, 2H), 6.27 (d, *J* = 8.5 Hz, 2H), 3.34 (dd, *J* = 13.8, 6.8 Hz, 8H), 2.32 (s, 3H), 1.17 (t, *J* = 6.9 Hz, 12H). ^{13}C NMR (100 MHz, $CDCl_3$, TMS): δ (ppm) = 165.2, 152.7, 152.3, 150.0, 148.9, 138.9, 138.6, 136.4, 136.0, 134.9, 133.4, 131.0, 130.5, 129.5, 128.3, 126.8, 126.3, 125.5, 123.4, 108.1, 105.6, 97.9, 65.7, 44.3, 21.3, 19.8, 12.6. IR (cm^{-1}): 3014.67, 2969.42, 2931.73, 1688.99, 1615.08, 1514.79, 1465.73, 1426.36, 1399.96, 1305.46, 1265.07, 1220.03, 1118.46, 1017.39, 975.00, 866.26, 819.99, 785.78, 754.50, 699.49, 683.62.

2. Spectroscopic properties of **1a-1i**

All probes (**1a-1i)** exhibited similar spectroscopic properties upon binding Cu^{2+}. The substituent moieties had effects on both the fluorescence and absorption intensities of the probes. However, no effects were found to be caused by the R moiety on the other spectral properties of the probes. The absorption and fluorescence emission wavelengths of these probes all appeared at 556 nm and 576 nm, respectively. Good linear relationships were observed between the relative fluorescence intensities of the probes and the concentration of Cu^{2+} in the 3-50 μM range in ethanol-PBS (5/5, v/v, pH 7.4) solution using 10μM of the respective probes. Optical spectra of each probe are shown as follow:

The solvent ratio (EtOH-H_2O=5:5), we chose, is an optimal choice which was gained from solvent selection experiment that contains seven binary solvents (organic solvent-H_2O = 5:5, organic solvent = EtOH, MeOH, $(CH_3)_2CHOH$, MeCN, THF, DMSO, DMF). We take probe **1a** as representative (**Figure R1)**, the result indicated that of MeOH-H_2O = 5:5 and EtOH-H_2O = 5:5 have better response than other mixed systems, so EtOH-H_2O was chosen to carry these optical experiments in consideration of the high toxicity of MeOH.

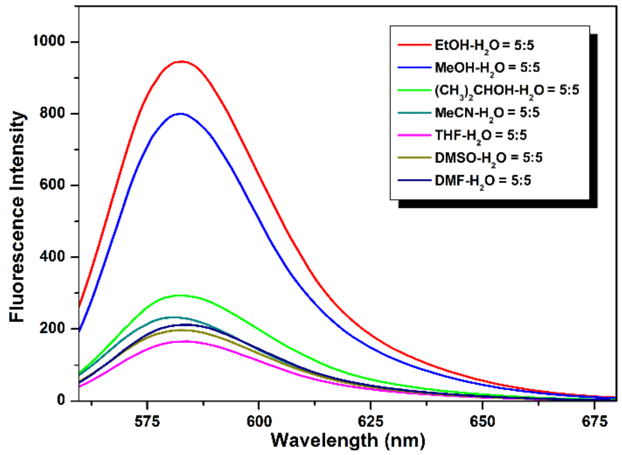

Figure S1 Fluorescence intensity of probe **1a** + Cu^{2+}(5.0 equiv.) in different binary solvents

Also, we have investigated Fluorescence intensity of probes **1a-1i** via EtOH-H_2O in varying proportions, and the results are showed in **Figure S2**. It is clearly observed that the response is weakest when pure water was used, and gradually increased along with the augment of ethanol proportion. When the ratio reached 5:5, the increasing trend weakens rapidly and the fluorescence intensity increases no more. As we all know, it's better to get best properties using organic solvent

as little as possible for the application in living system, so EtOH-H_2O=5:5 is the best choice that we have made for all the optical test.

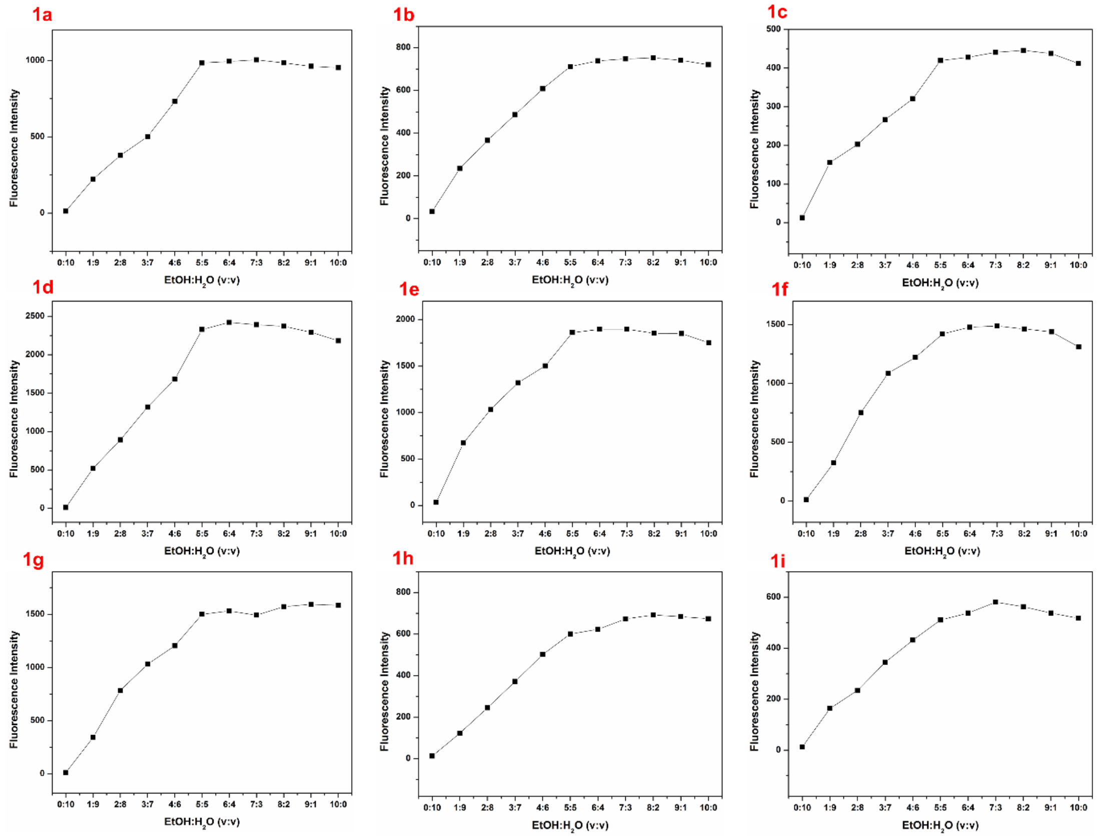

Figure S2. Fluorescence intensity of probe **1a-1i** + Cu^{2+}(5.0 equiv.) in EtOH-H_2O binary solvent of varying proportions.

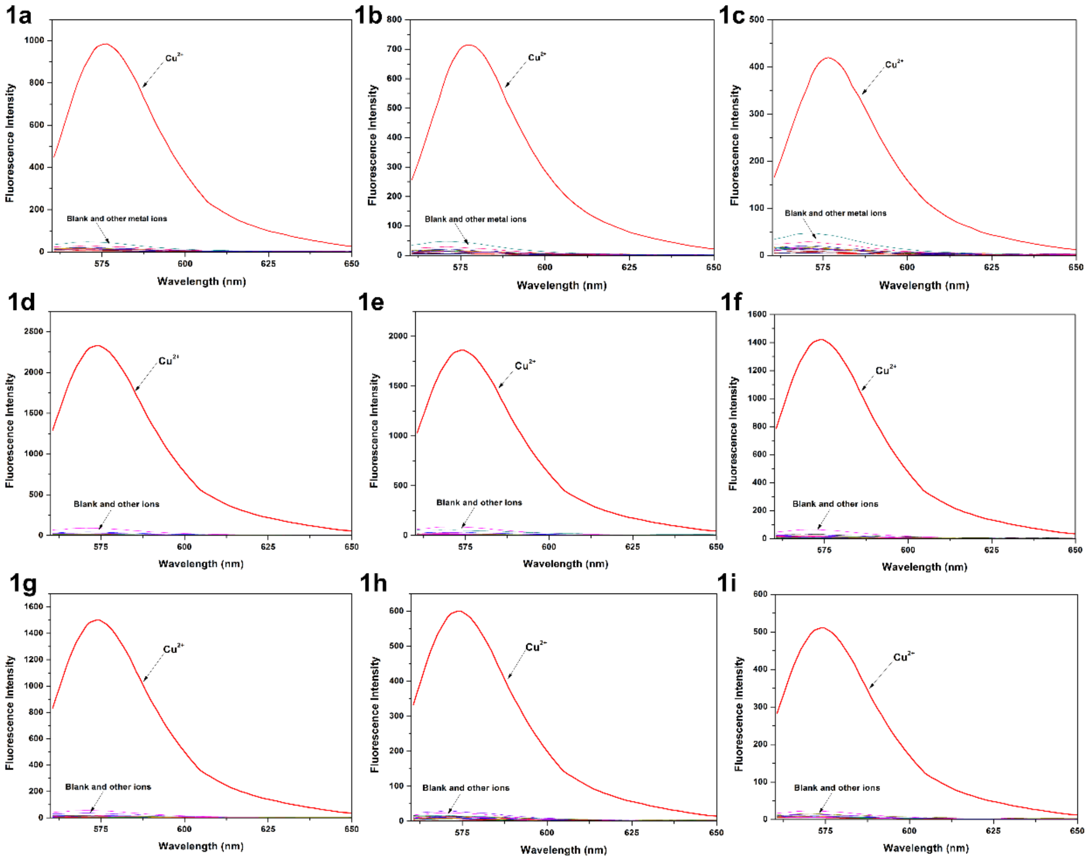

Fig. S3 Fluorescence spectra of **1a-1i** (10 μM) in ethanol-PBS (5/5, v/v, pH 7.4) solution upon addition of various metal ions (50 μM), λex = 550 nm.

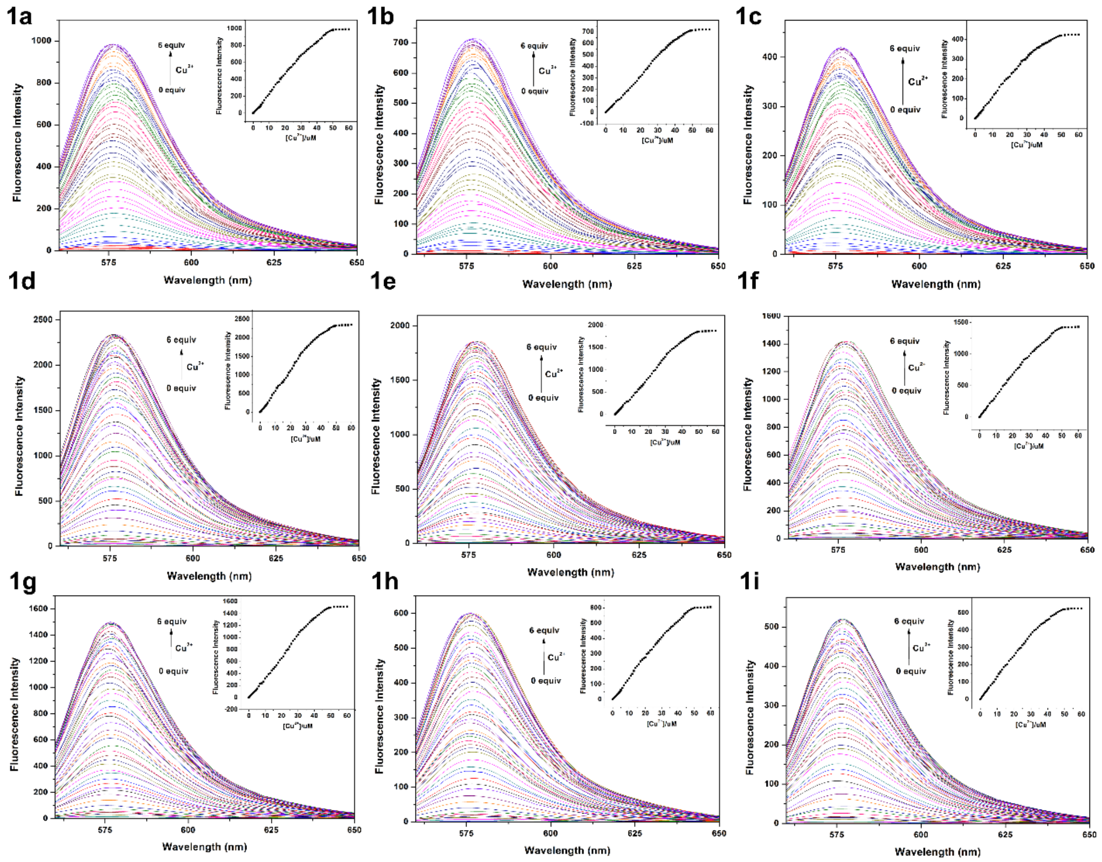

Fig. S4 Fluorescence intensity changes of **1a-1i** (10 μM) upon addition of Cu^{2+} (0-6.0 equiv) in ethanol-PBS (5/5, v/v, pH 7.4) solution, λex = 550 nm. Inset: Changes in the emission intensity at 576 nm.

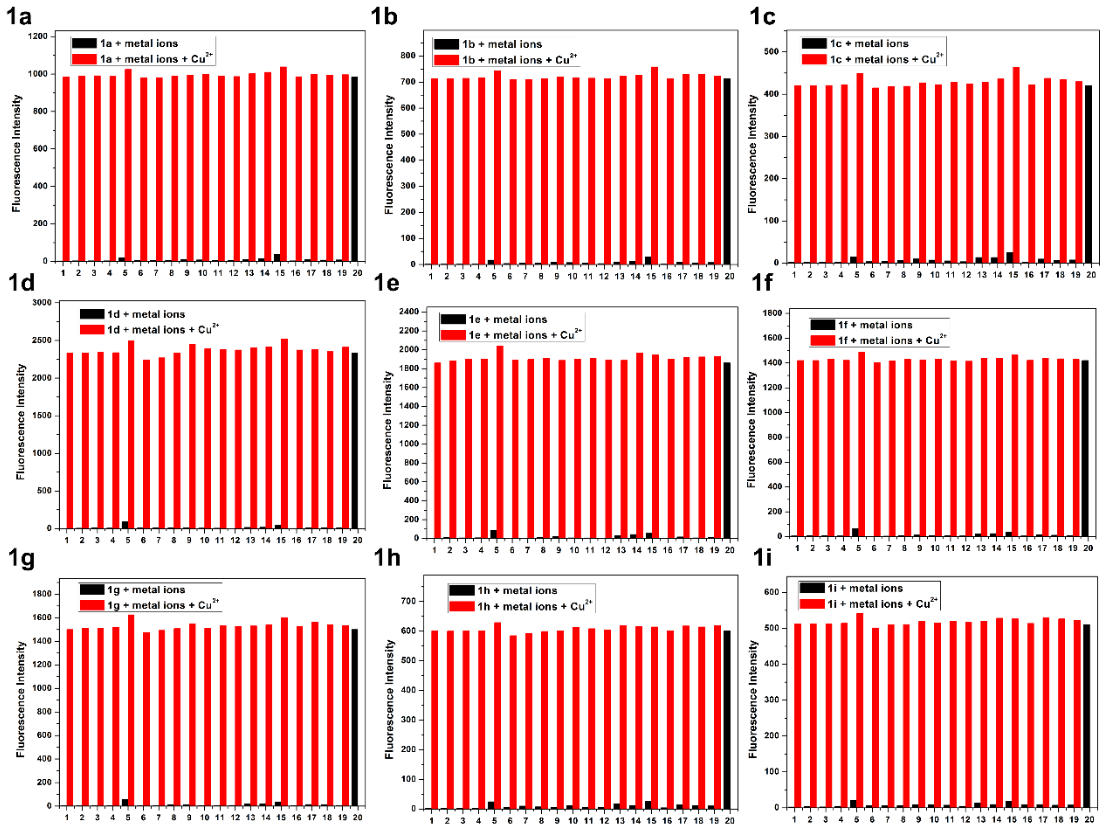

Fig. S5 Fluorescence intensity changes of **1a-1i** (10 μM) upon the addition of various metal ions (50 μM) in and without the presence of Cu^{2+} (50 μM) in ethanol-PBS (5/5, v/v, pH 7.4) solution. Black bars represent the fluorescence response of **1a-1i** to the metal ions of interest. 1, blank; 2, Li^{+}; 3, Na^{+}; 4, K^{+}; 5, Ag^{+}; 6, Ba^{2+}; 7, Ca^{2+}; 8, Mg^{2+}; 9, Cd^{2+}; 10, Mn^{2+}; 11, Co^{2+}; 12, Fe^{2+}; 13, Ni^{2+}; 14, Zn^{2+}; 15, Pb^{2+}; 16, Hg^{2+}; 17, Fe^{3+}; 18, Cr^{3+}; 19, Al^{3+}; 20, Cu^{2+}. The chromatic bars represent the subsequent addition of 50 μM Cu^{2+} to the above solutions.

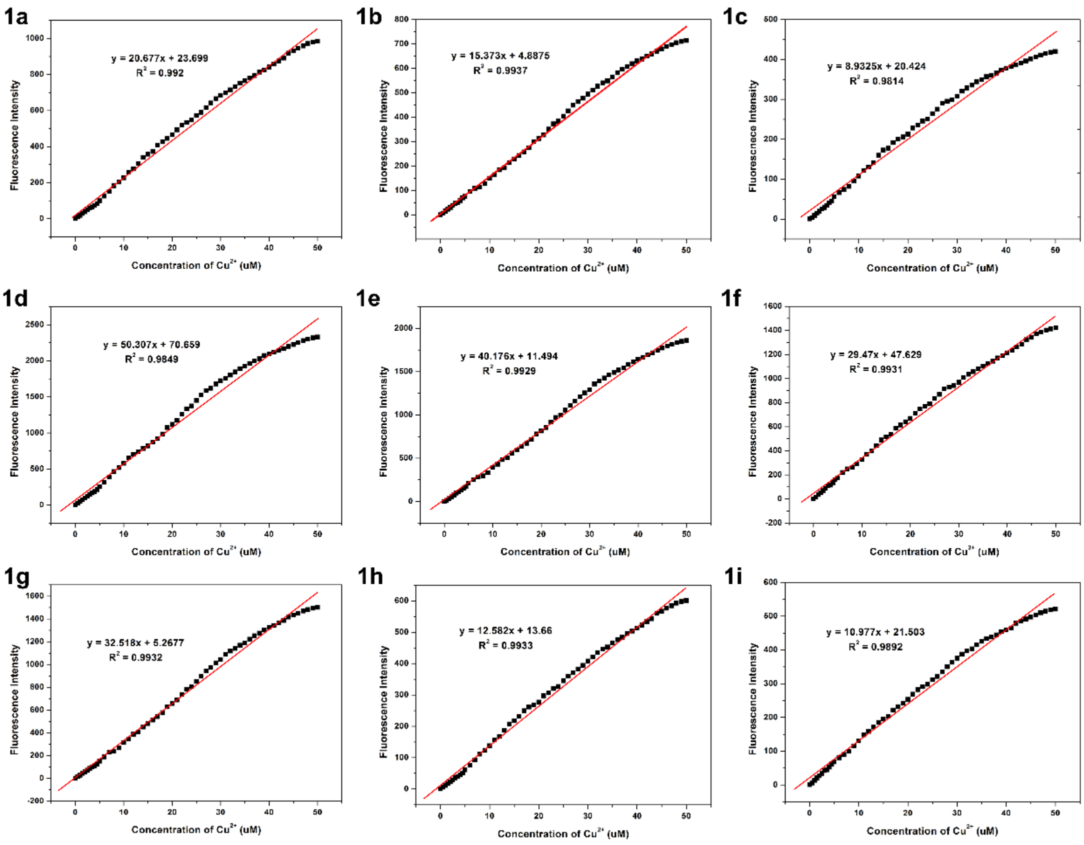

Fig. S6 Fluorescence intensity changes of **1a-1i** in the emission intensity at 576 nm.

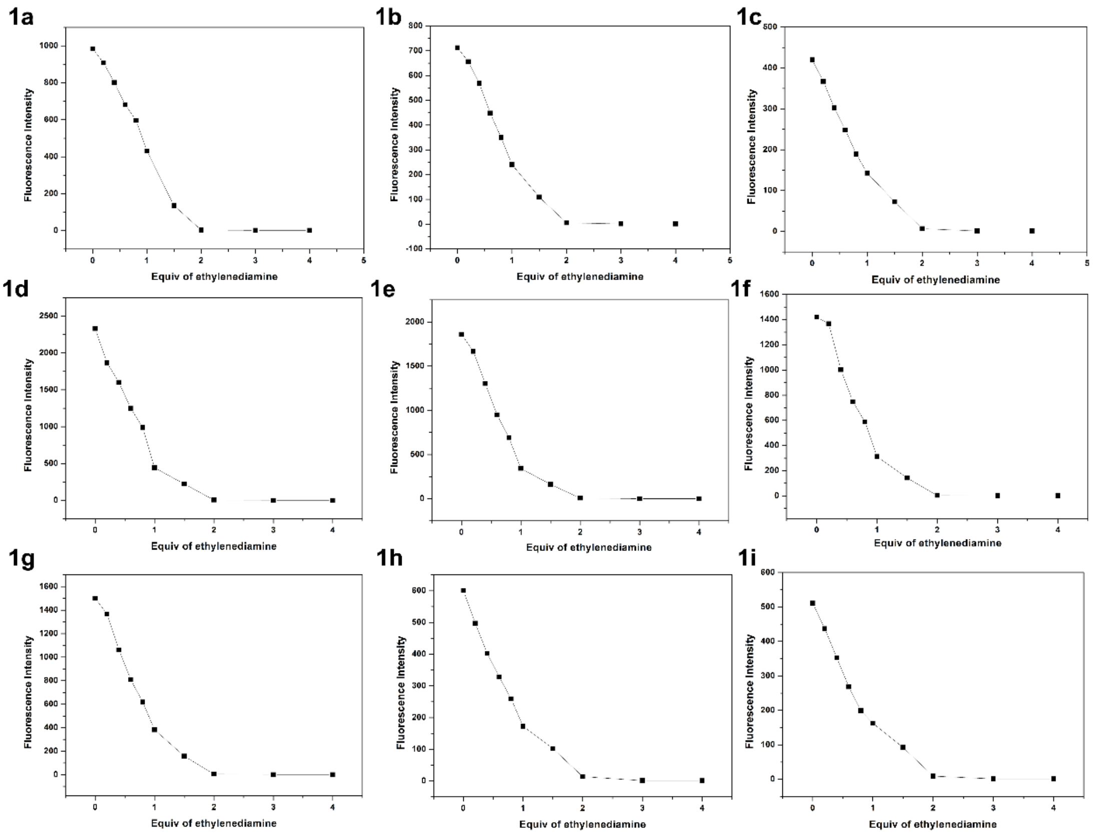

Fig. S7 Fluorescence intensity changes of **1a-1i** (10 μM) upon the addition of each equiv of ethylenediamine with the presence of Cu^{2+} (50 μM) in ethanol-PBS (5/5, v/v, pH 7.4) solution, λex = 550 nm.

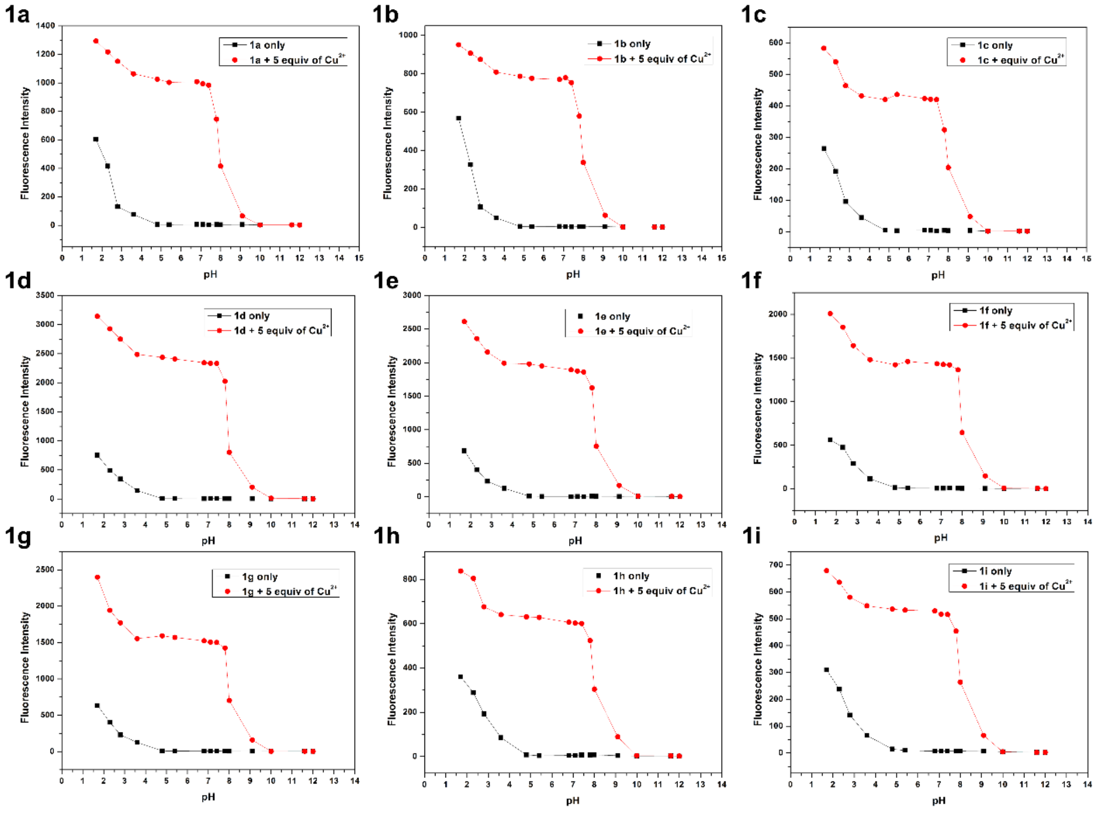

Fig. S8 Fluorescnece intensity of **1a-1i** (10 μM) in the absence and presence of 5.0 equiv of Cu^{2+} in ethanol-water (5/5, v/v) solution with different pH conditions, λex = 550 nm.

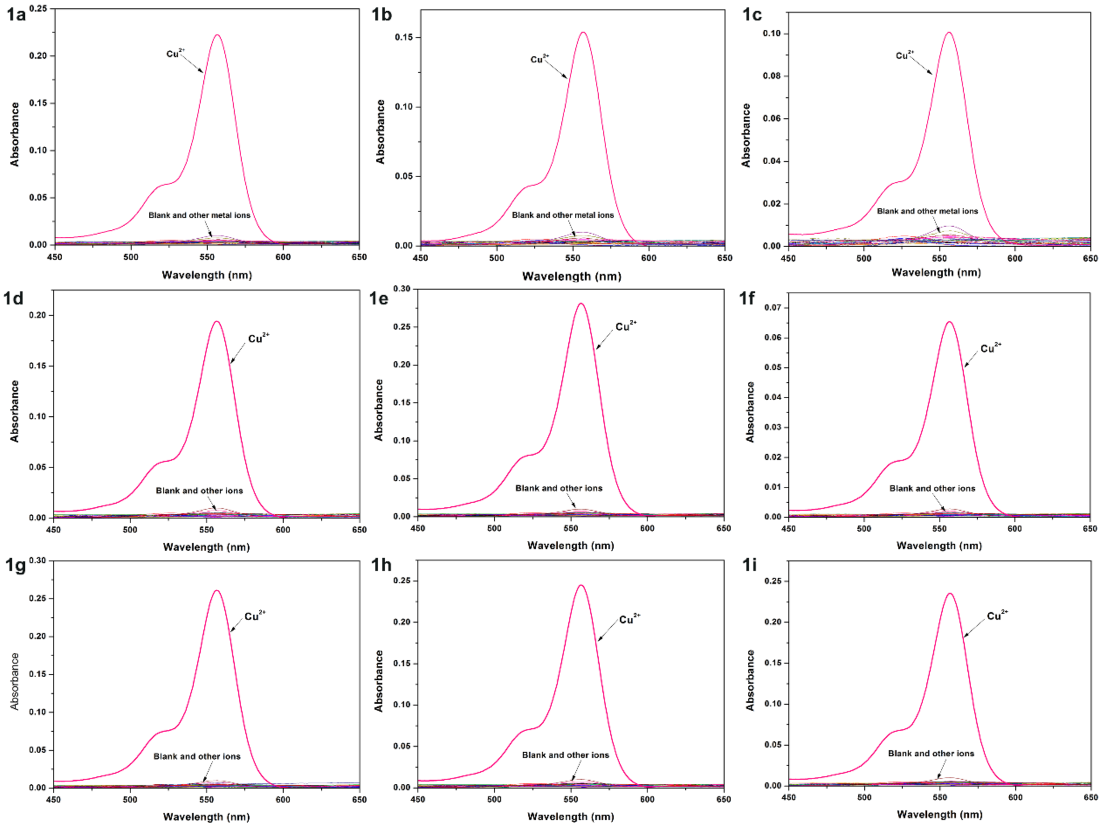

Fig. S9 Absorption spectra of **1a-1i** (10 μM) in ethanol-PBS (5/5, v/v, pH 7.4) solution upon addition of various metal ions (50 μM).

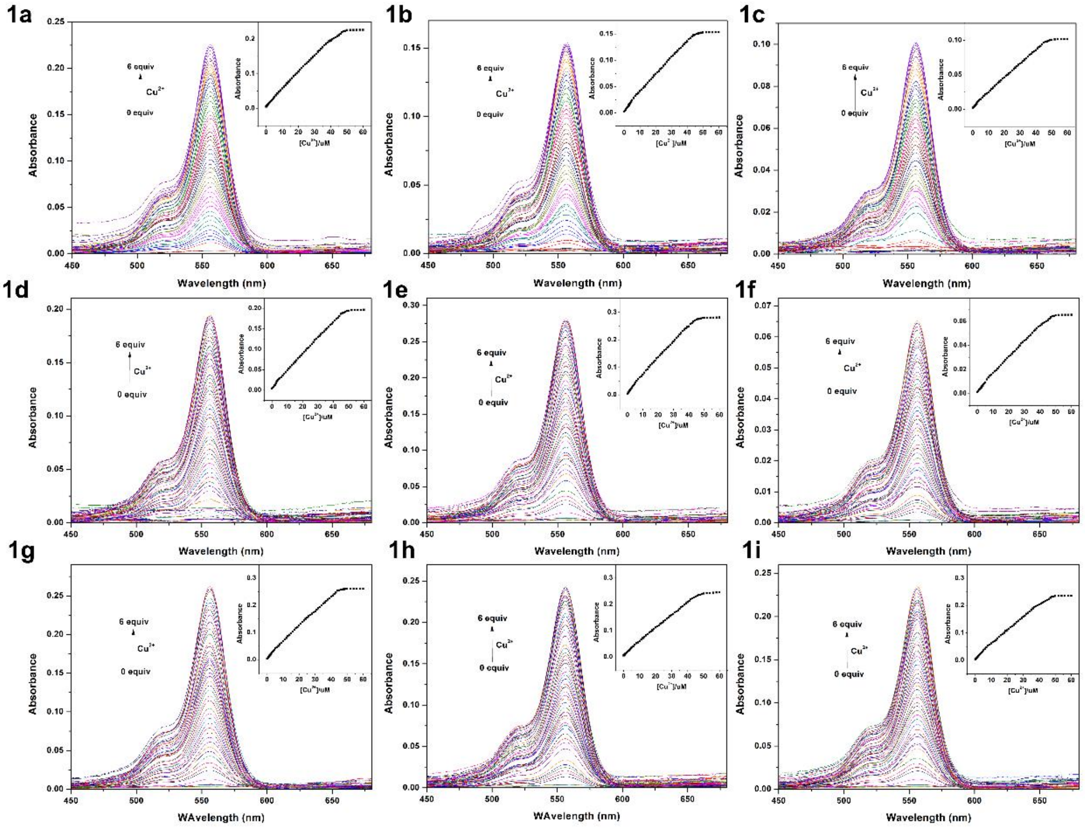

Fig. S10 Absorption changes of **1a-1i** (10 μM) upon addition of Cu^{2+} (0-5.0 equiv) in ethanol-PBS (5/5, v/v, pH 7.4) solution. Inset: Absorbance at 556 nm of **1a-1i** as a function of Cu^{2+} concentration.

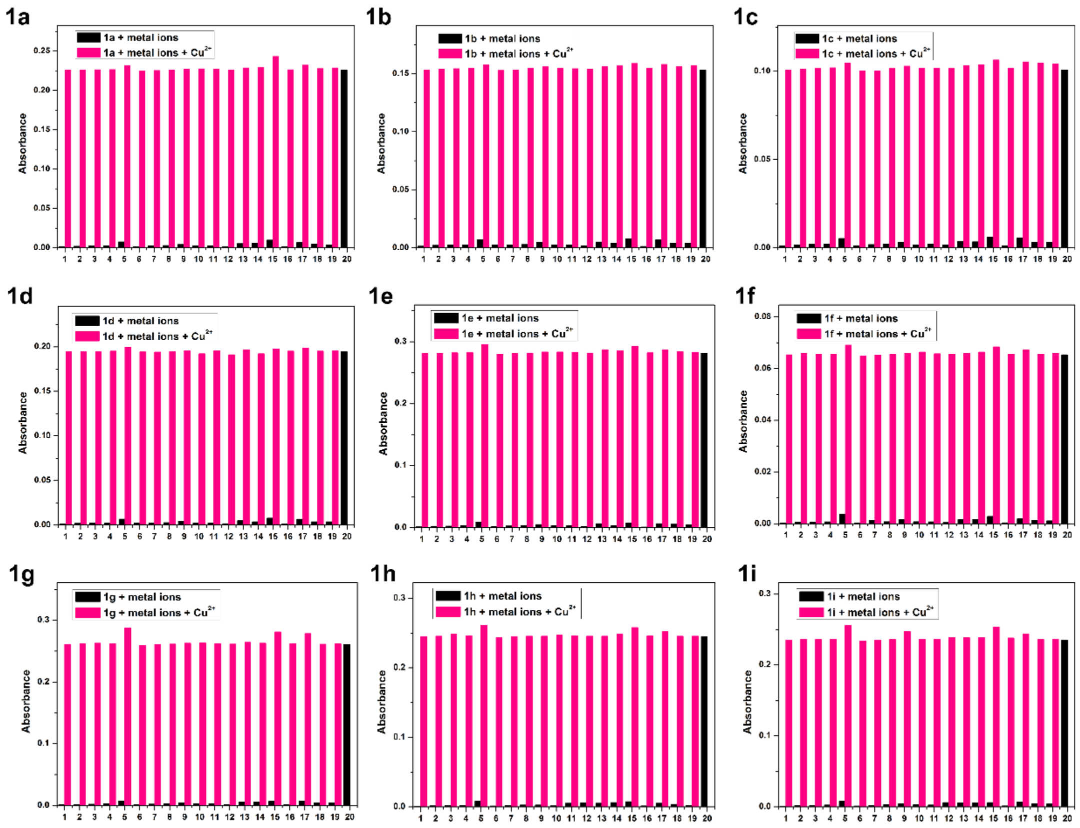

Fig. S11 Absorption changes of **1a-1i** (10 μM) upon the addition of various metal ions (50 μM) in and without the presence of Cu^{2+} (50 μM) in ethanol-PBS (5/5, v/v, pH 7.4) solution. Black bars represent the fluorescence response of **1a-1i** to the metal ions of interest. 1, blank; 2, Li^{+}; 3, Na^{+}; 4, K^{+}; 5, Ag^{+}; 6, Ba^{2+}; 7, Ca^{2+}; 8, Mg^{2+}; 9, Cd^{2+}; 10, Mn^{2+}; 11, Co^{2+}; 12, Fe^{2+}; 13, Ni^{2+}; 14, Zn^{2+}; 15, Pb^{2+}; 16, Hg^{2+}; 17, Fe^{3+}; 18, Cr^{3+}; 19,Al^{3+}; 20, Cu^{2+}. The chromatic bars represent the subsequent addition of 50 μM Cu^{2+} to the above solutions.

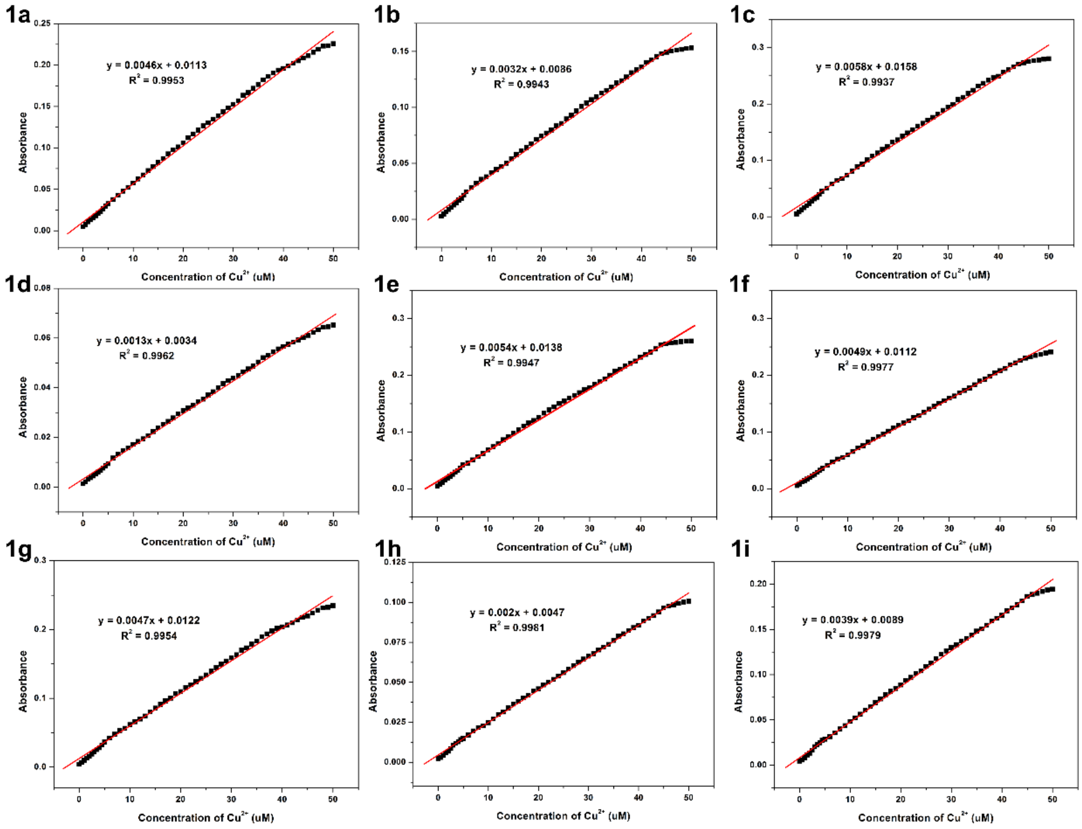

Fig. S12 Absorbance at 556 nm of **1a-1i** as a function of Cu^{2+} concentration.

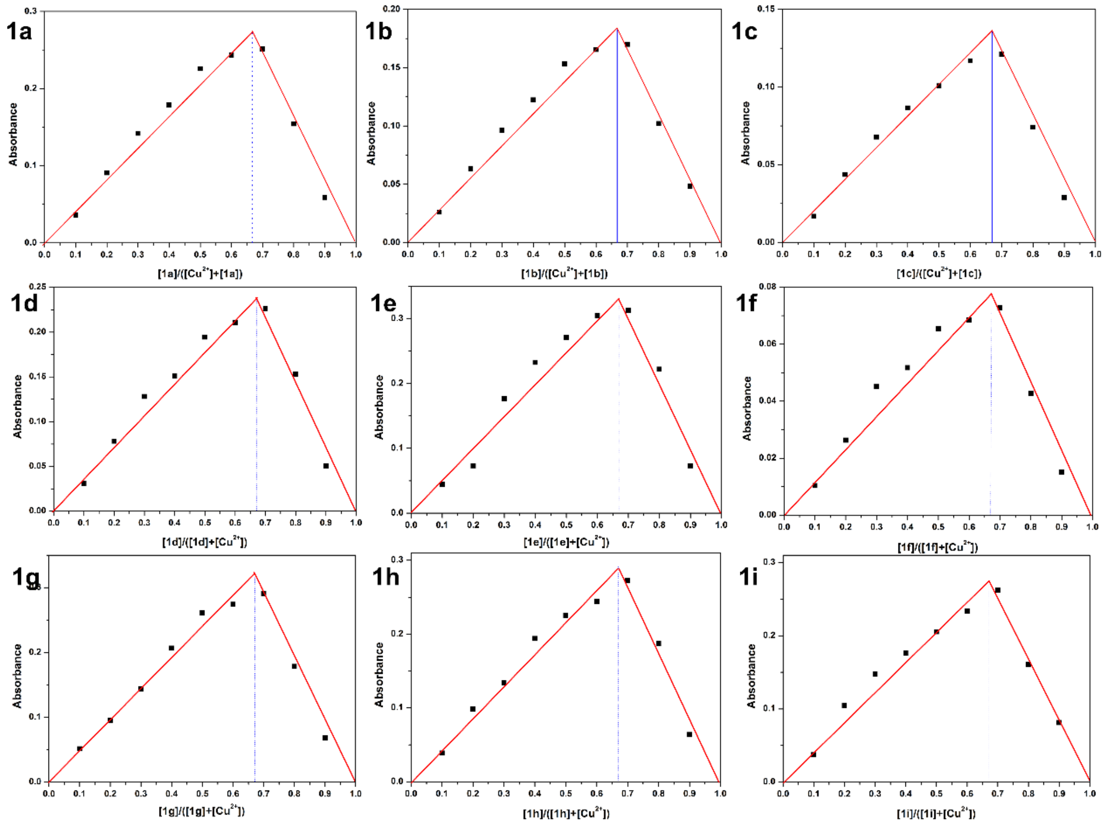

Fig. S13 Job's plot of **1a-1i** and Cu^{2+}. The total concentration of **1a-1i** and Cu^{2+} was 20 μM. The absorbance was measured at 556 nm.

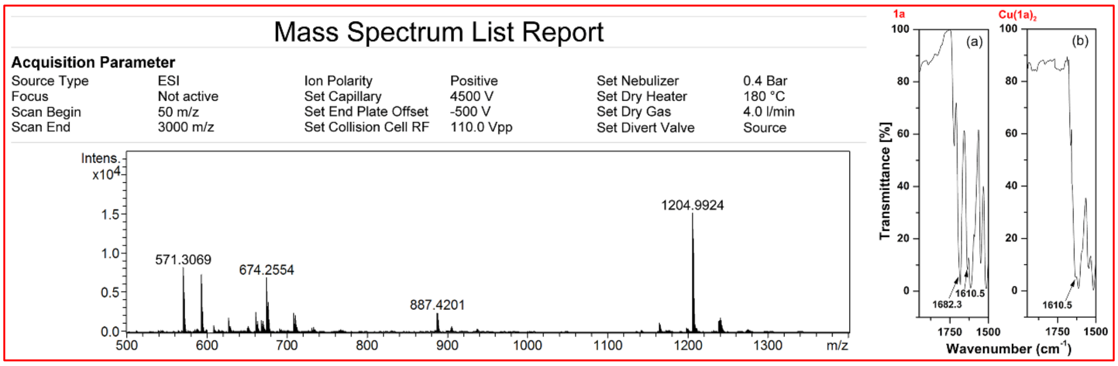

Figure S14. HRMS of $Cu(\mathbf{1a})_2$ and IR of **1a**/ $C(\mathbf{1a})_2$

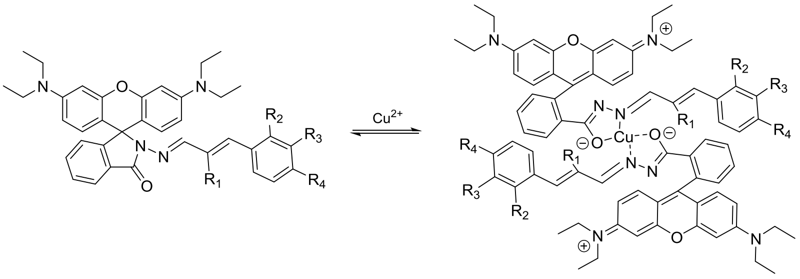

Figure S15. Proposed mechanism for the fluorescent changes upon the addition of Cu^{2+}.

3. Equations used for the calculation of association constant about **1a-1i**

The association constants were determined according to the references[1-3]. The sensors bind with Cu^{2+} forming the 1:2 metal-ligand complex between Cu^{2+} and the sensors, the equilibrium can be described as follows:

$$R + Cu^{2+} \underset{}{\overset{K}{\rightleftharpoons}} CuR_2 \qquad (3\text{-}1)$$

Here, R and CuR_2 denote the probes **1a-1i** and the complex, respectively, and K denotes the association constant. The corresponding association constant ***K*** can be expressed as follows:

$$K = \frac{[CuR_2]}{[R][Cu^{2+}]} \qquad (3\text{-}2)$$

The relative absorbance R is defined as the ratio of free R. $[R]_f$ is defined as the total amount of R. $[R]_t$ is defined as the absorbance in the ethanol-PBS (5/5, v/v, pH 7.4) solution. It can be experimentally determined by measuring the absorbance values in the presence of different concentrations of Cu^{2+}

$$\alpha = \frac{[R]_f}{[R]_t} = \frac{A - A_0}{A_t - A_0} \qquad (3\text{-}3)$$

Here, A_0 and A_t are the limiting absorbance values for $\alpha=1$ (in the absence of Fe^{3+}) and $\alpha=0$ (the probe is completely complexed with Cu^{2+}), respectively. The relationship between the probe and the Cu^{2+} concentration can be represented as follows:

$$\frac{\alpha^2}{1 - \alpha} = \frac{1}{2K[R]_t[Cu^{2+}]} \qquad (3\text{-}4)$$

It is apparent from Equation 3-4 that the relative absorbance α has a distinct functional relationship with the concentration of Cu^{2+} and the association constant K, which provides the basis for the detection of the K value. The experimental data were fitted to Equation 3-4 by adjusting the K value.

The association constants of probe **1a-1i** with copper ion respectively were determined to be: **1a**, 8.90×10^4 M^{-1}; **1b**, 1.26×10^4 M^{-1}; **1c**, 7.20×10^4 M^{-1}; **1d**,1.08×10^5 M^{-1}; **1e**, 1.25×10^5 M^{-1}; **1f**, 4.09×10^4 M^{-1}; **1g**, 2.92×10^5 M^{-1}; **1h**, 1.05×10^5 M^{-1}; **1i**, 4.21×10^4 M^{-1}, it can be found that, as a whole, large association constant indicates good optical performance in this modular Cu^{2+} fluorescent probes.[4-6] For probe **1a-1c**, **1a** has the largest association constants than the remaining two probes,

which is coincident with their fluorescence and absorption order and may be caused by the smallest steric hindrance of **1a**.; Probe **1d** shows a high association constant, which may be one reason that leads to its highest fluorescence intensity and relatively stronger absorption strength over other probes; Probe **1e** shows higher association constant than that of **1f**, which is coincident with their fluorescence and absorption order and may attributed to the strong electron-withdrawing of nitryl; For probe **1g-1i**, the order association constants is consistent with that of their fluorescence intensity and absorption strength.

4. Equations used for the calculation of fluorescence quantum yield

Fluorescence quantum yields (Φ) were determined using optically matching solutions of rhodamine B (Φf = 0.97 in ethanol) as standards at an excitation wavelength of 558 nm and the quantum yields were calculated using Equation according to the references[7,8]. Where *Φu* and *Φs* are the fluorescence quantum yields of the sample and standard, *Iu* and *Is* are the integrated emission intensities of the corrected spectra for the sample and standard, *Au* and *As* are the absorbance of the sample and standard at the excitation wavelength (556 nm in all cases), and *ηu* and *ηs* are the indices of refraction of the sample and standard solutions, respectively.

$$\Phi_u = \Phi_s \cdot \frac{I_u}{I_s} \cdot \frac{A_s}{A_u} \cdot \left(\frac{\eta_u}{\eta_s}\right)^2 \qquad (4\text{-}1)$$

The fluorescence quantum yields of **1a-1i** were determined to be: **1a**, 0.54; **1b**, 0.51; **1c**, 0.44. **1d**, 0.73. **1e**, 0.65. **1f**, 0.89. **1g**, 0.61. **1h**, 0.27. **1i**, 0.24. In general, the fluorescence quantum yields of this modular probes **1a-1i** are basically consistent with their fluorescence intensity. And because the fluorescence quantum yield shows not only a positive correlation with fluorescence intensity but also a negative correlation with absorption strength under the same circumstances (equation 4-1), so probe **1f** has the highest quantum yield over other probes due to its lowest absorption strength and the quantum yield of probe **1c** is higher than that of **1h** and **1i**, which may be also caused by the much lower absorption strength of **1c** has over **1h** and **1i**.

5. Calculation of the detection limit of probe **1a-1i**

$$\mathbf{LOD = 3\sigma/k} \qquad (5\text{-}1)$$

Where, σ is the standard deviation of the blank solution and k is the slope of the linear calibration plot between the fluorescence intensity/absorption and the concentration of Cu^{2+}. The calculated LOD of probe **1a-1i** are showed in **Table S1**.

Table S1. The calculated detection limit for probe **1a-1i**

	Probe	1a	1b	1c	1d	1e	1f	1g	1h	1i
**LOD**	FL/nM	270	409	596	116	130	204	206	428	520
	UV/nM	3094	3419	3824	3106	2652	4439	2794	28592	2925

The results show that, for fluorescence experiment, the order of LOD of this modular probes **1a-1i** is **1d** < **1e** < **1f** ≈ **1g** < **1a** < **1b** < **1h** < **1i** < **1c**, which indicates that the probe that exhibited a higher emission intensity almost have a lower detection limit. And in absorbance experiment, the LOD order of probe **1a-1i** is **1e** < **1g** < **1h** < **1i** < **1a** < **1d** < **1b** < **1c** < **1f**, also demonstrates that a higher absorption strength accompanies a lower LOD among this modular probes.[9]

6. IR, ^{1}H NMR, ^{13}C NMR and MS spectra of all the compounds

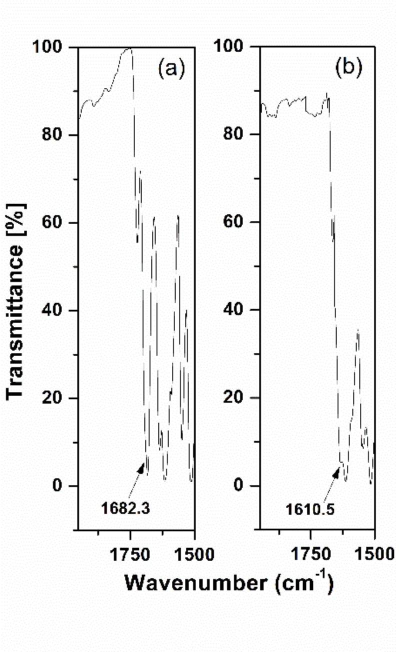

Fig. S16 IR spectra of compound **1a** (a) and Cu(**1a**)$_2$ complex (b) in KBr disks.

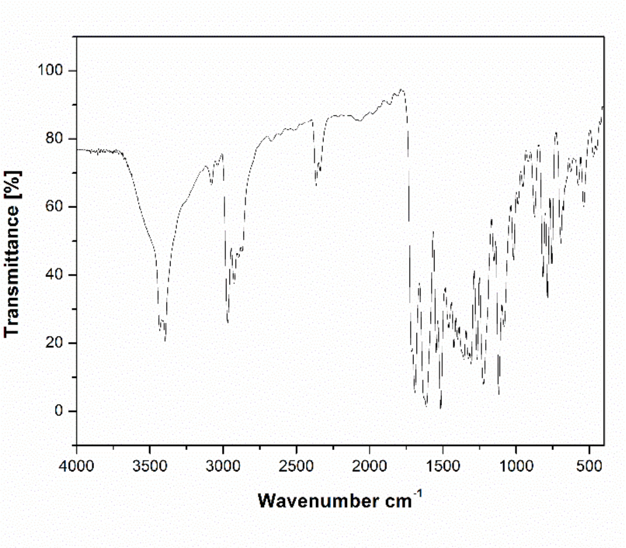

Fig. S17 IR spectrum of N-(rhodamine-B)lactam-hydrazine in KBr disks

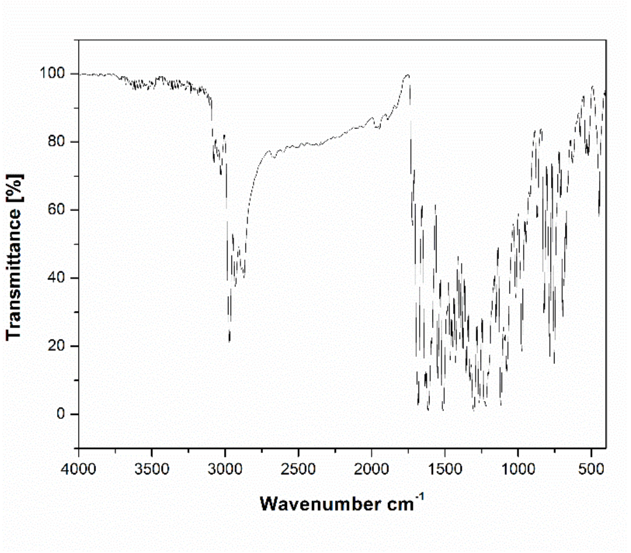

Fig. S18 IR spectrum of **1a** in KBr disks.

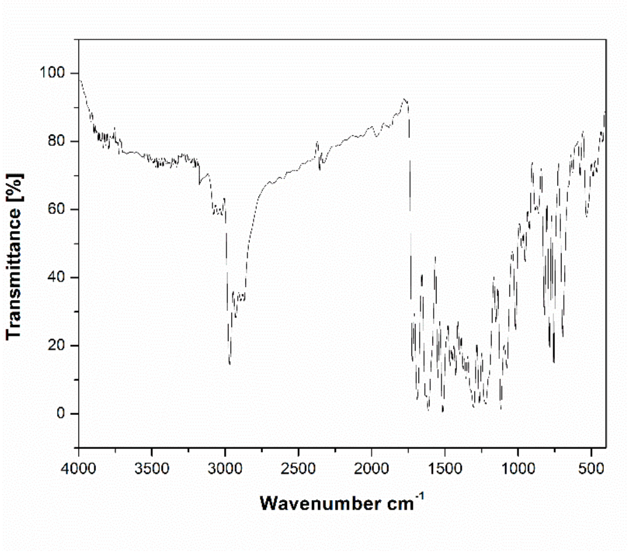

Fig. S19 IR spectrum of **1b** in KBr disks.

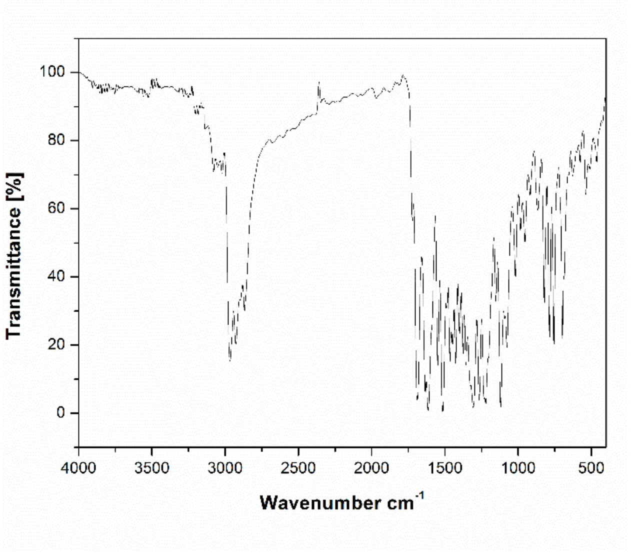

Fig. S20 IR spectrum of **1c** in KBr disks.

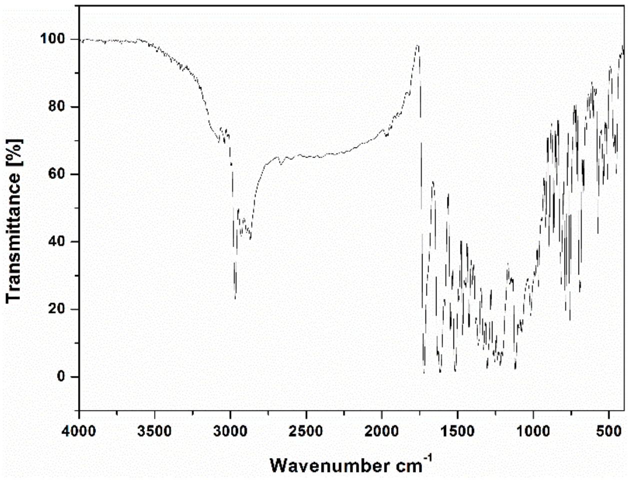

Fig. S21 IR spectrum of **1d** in KBr disks.

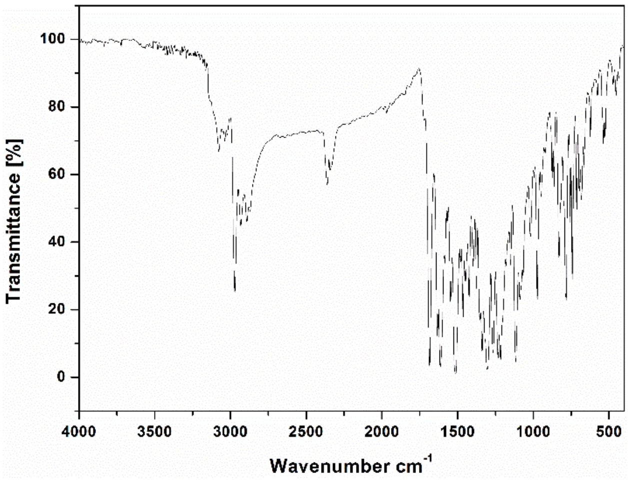

Fig. S22 IR spectrum of **1e** in KBr disks.

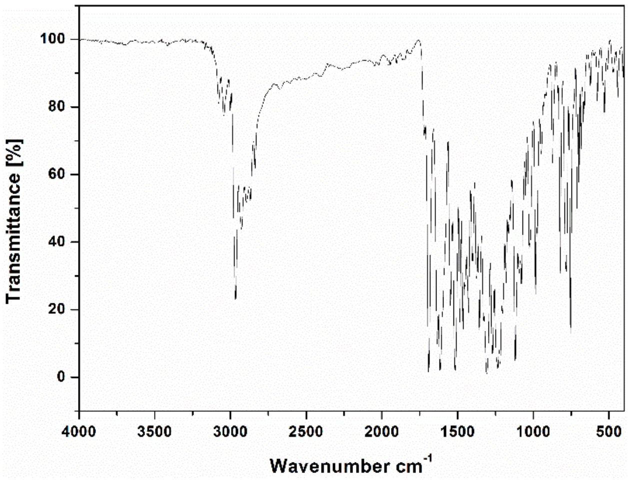

Fig. S23 IR spectrum of **1f** in KBr disks.

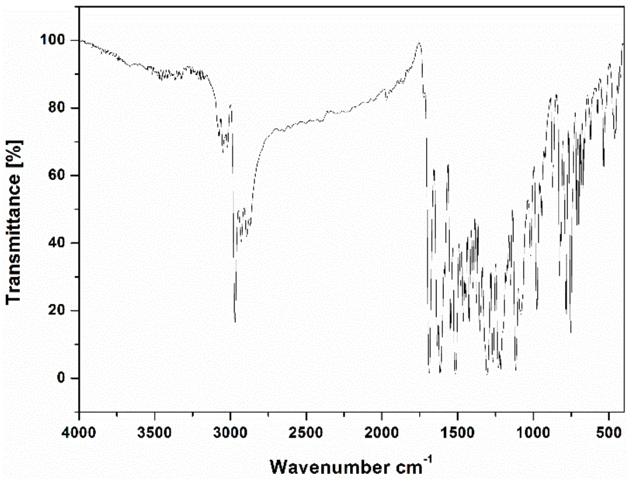

Fig. S24 IR spectrum of **1g** in KBr disks.

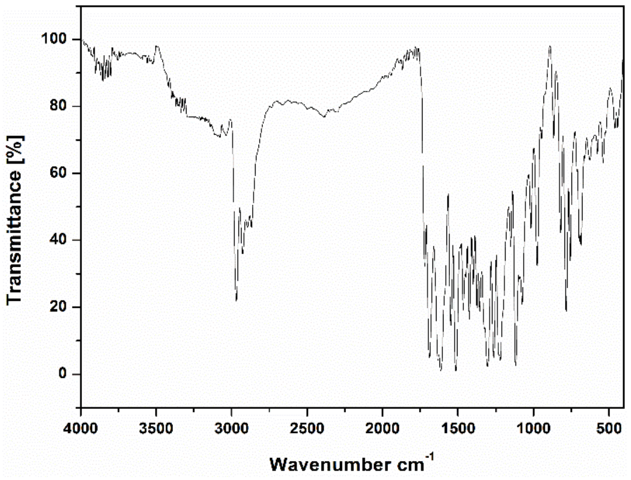

Fig. S25 IR spectrum of **1h** in KBr disks.

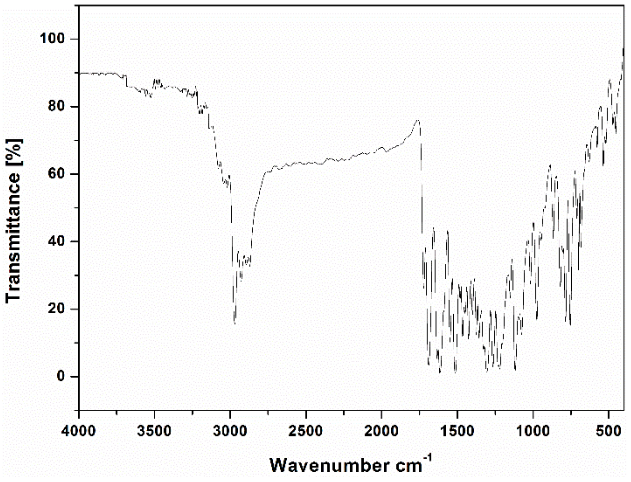

Fig. S26 IR spectrum of **1i** in KBr disks.

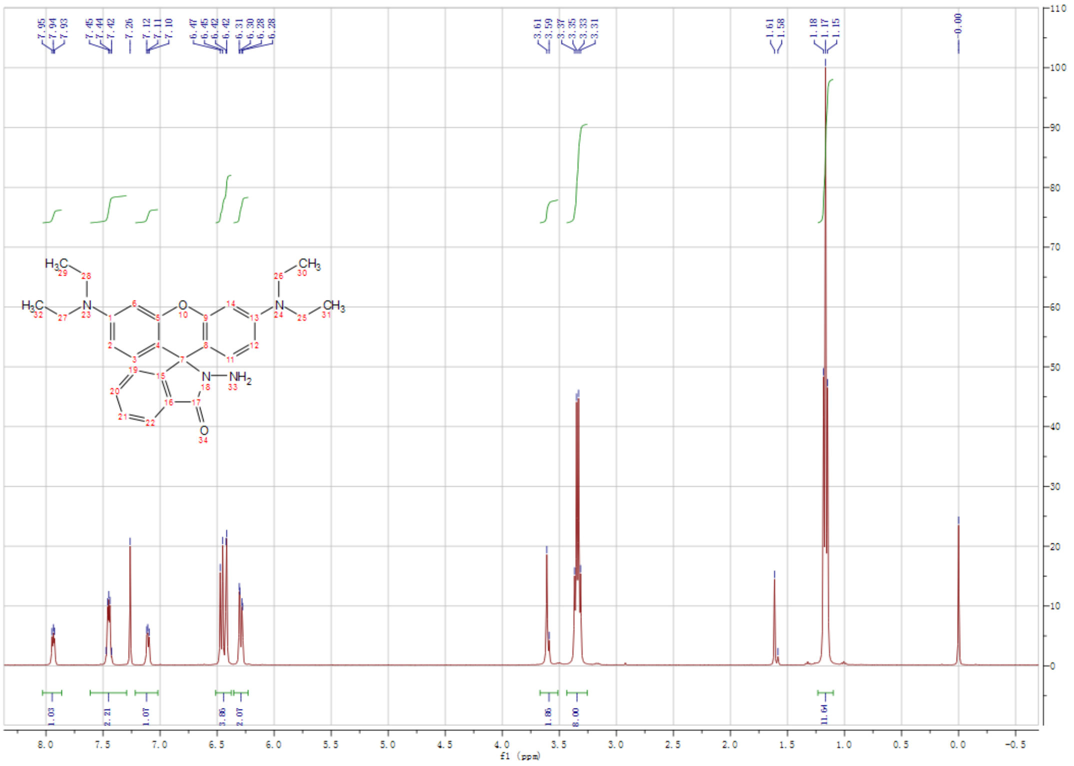

Fig. S27 ^{1}H NMR spectrum of N-(rhodamine-B)lactam-hydrazine in $CDCl_3$.

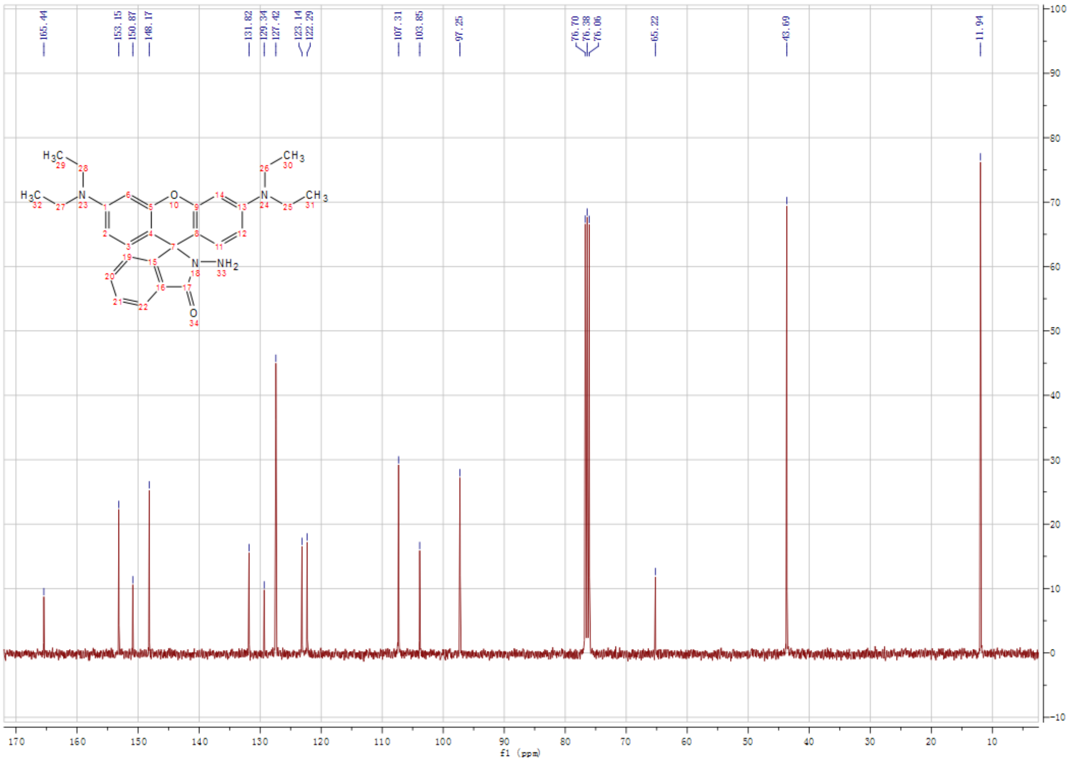

Fig. S28 ^{13}C NMR spectrum of N-(rhodamine-B)lactam-hydrazine in $CDCl_3$.

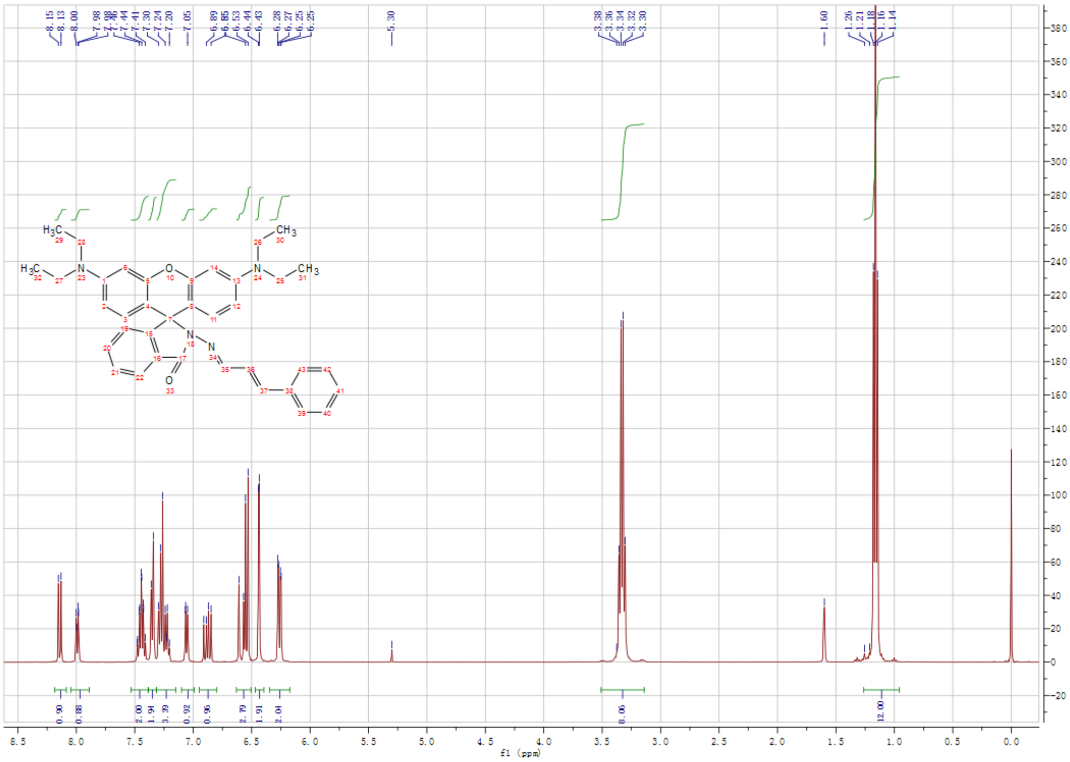

Fig. S29 ^{1}H NMR spectrum of **1a** in $CDCl_3$.

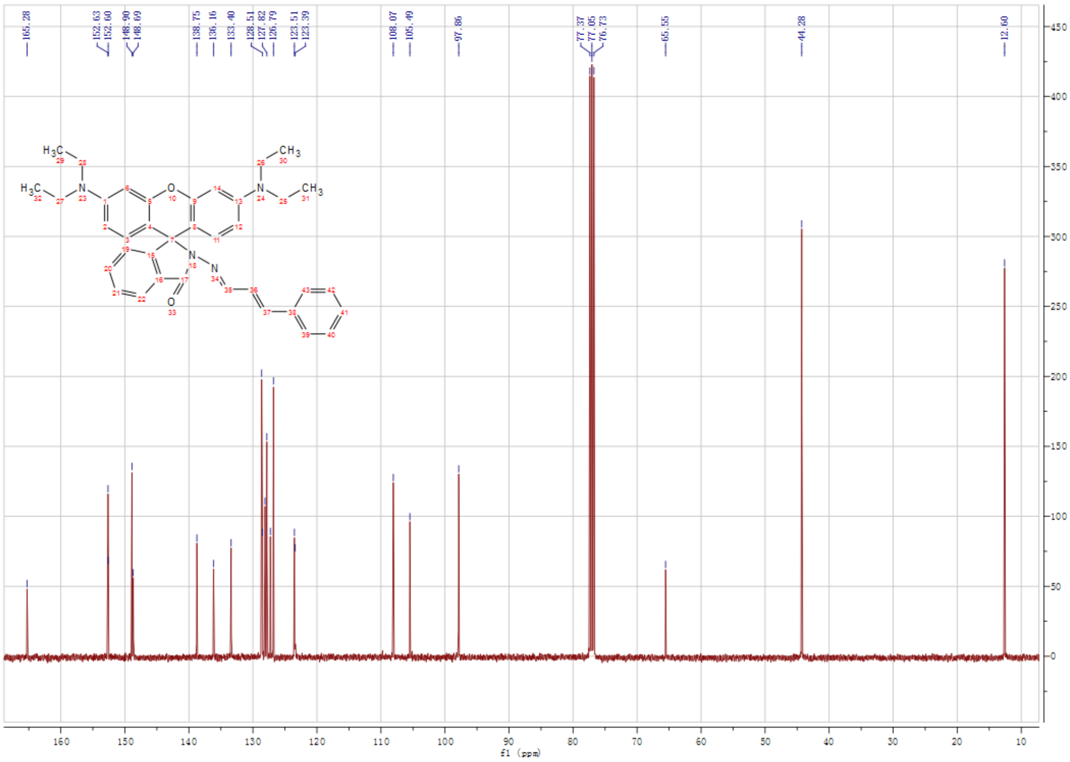

Fig. S30 ^{13}C NMR spectrum of **1a** in $CDCl_3$.

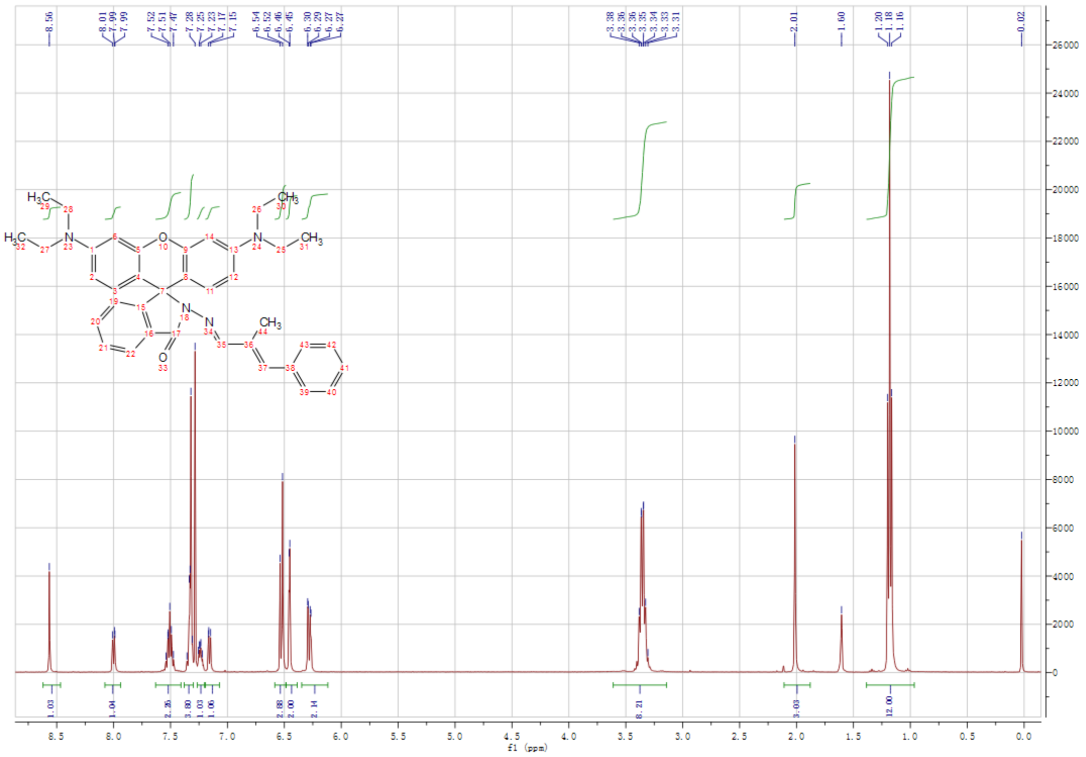

Fig. S31 ^{1}H NMR spectrum of **1b** in $CDCl_3$.

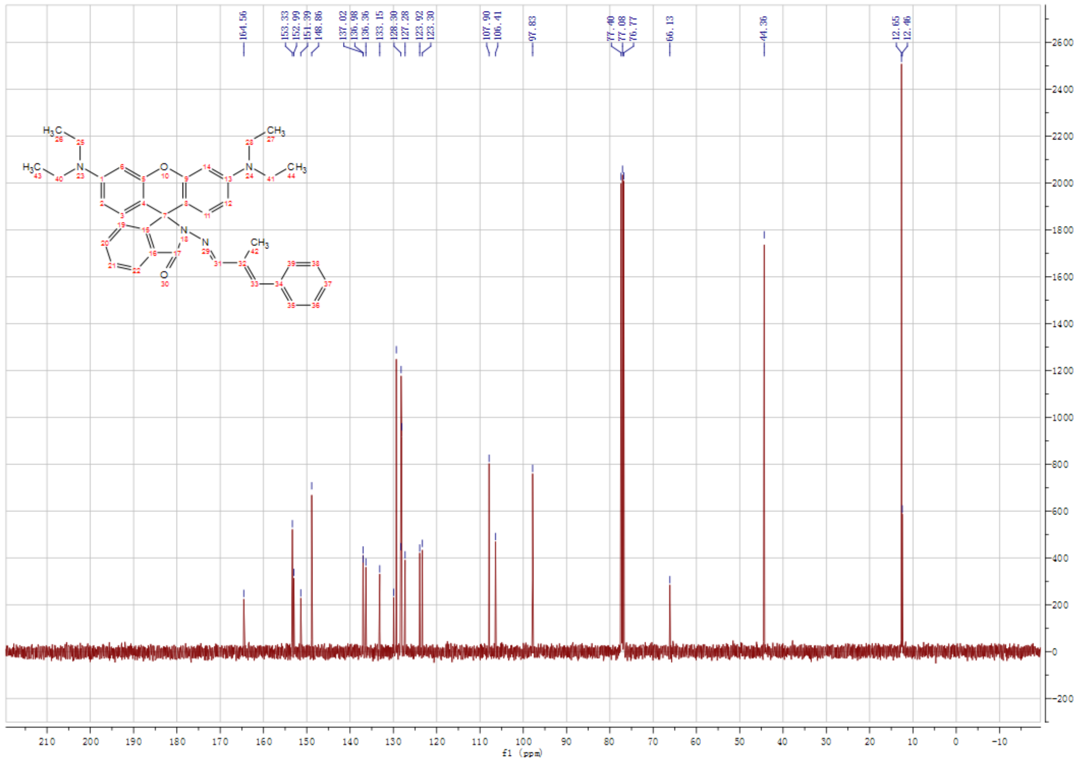

Fig. S32 ^{13}C NMR spectrum of **1b** in $CDCl_3$.

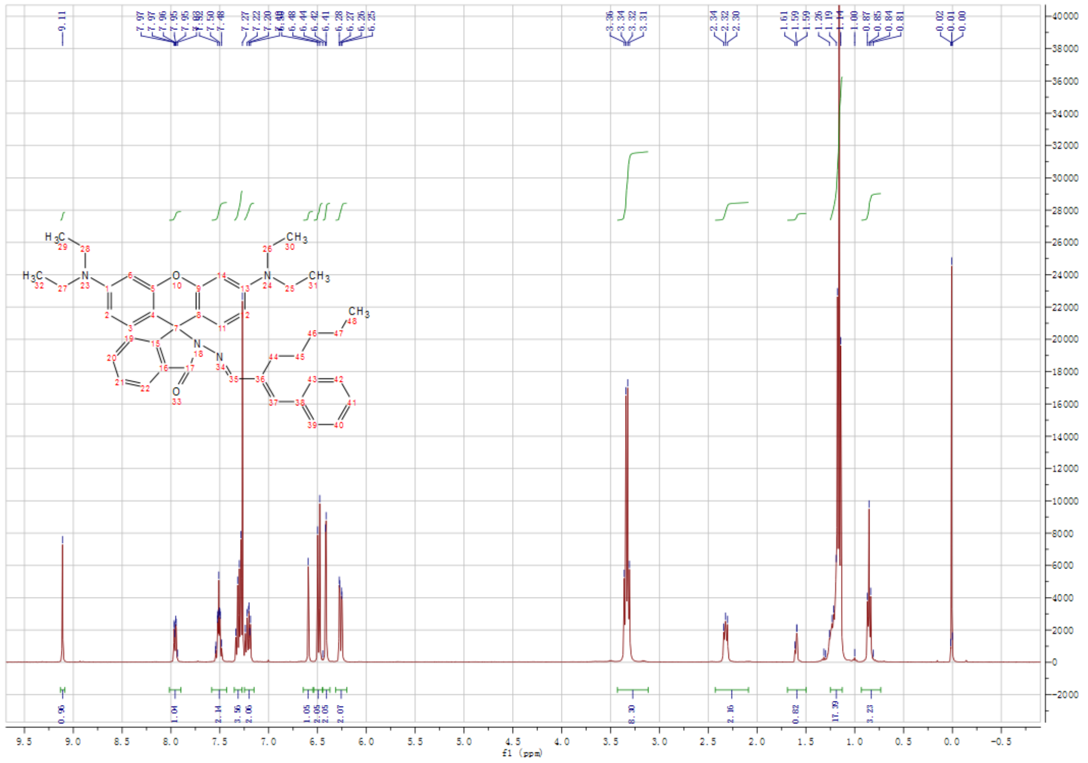

Fig. S33 ^{1}H NMR spectrum of **1c** in $CDCl_3$.

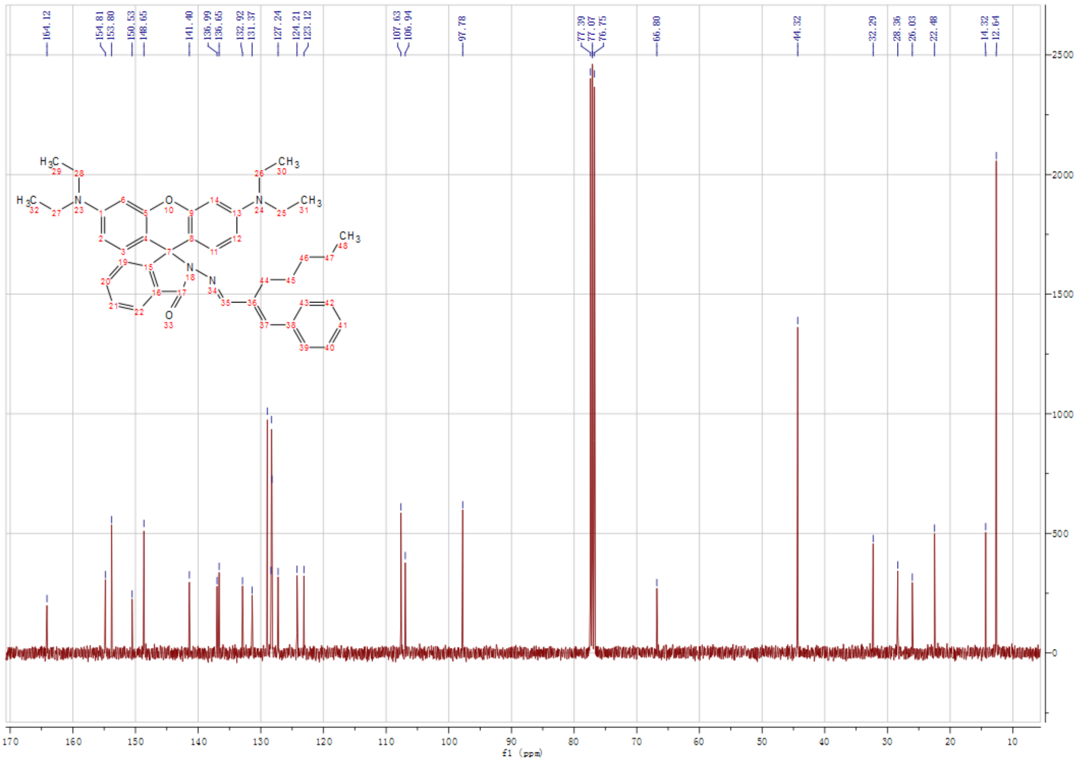

Fig. S34 ^{13}C NMR spectrum of **1c** in $CDCl_3$.

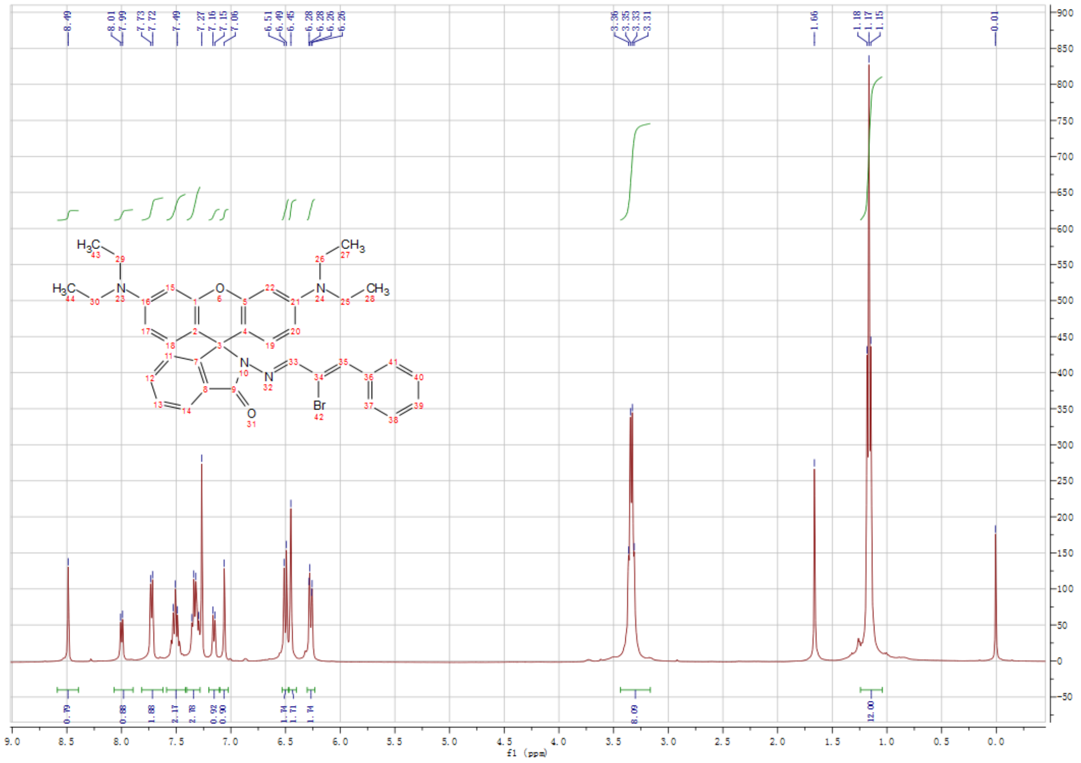

Fig. S35 ^{1}H NMR spectrum of **1d** in $CDCl_3$.

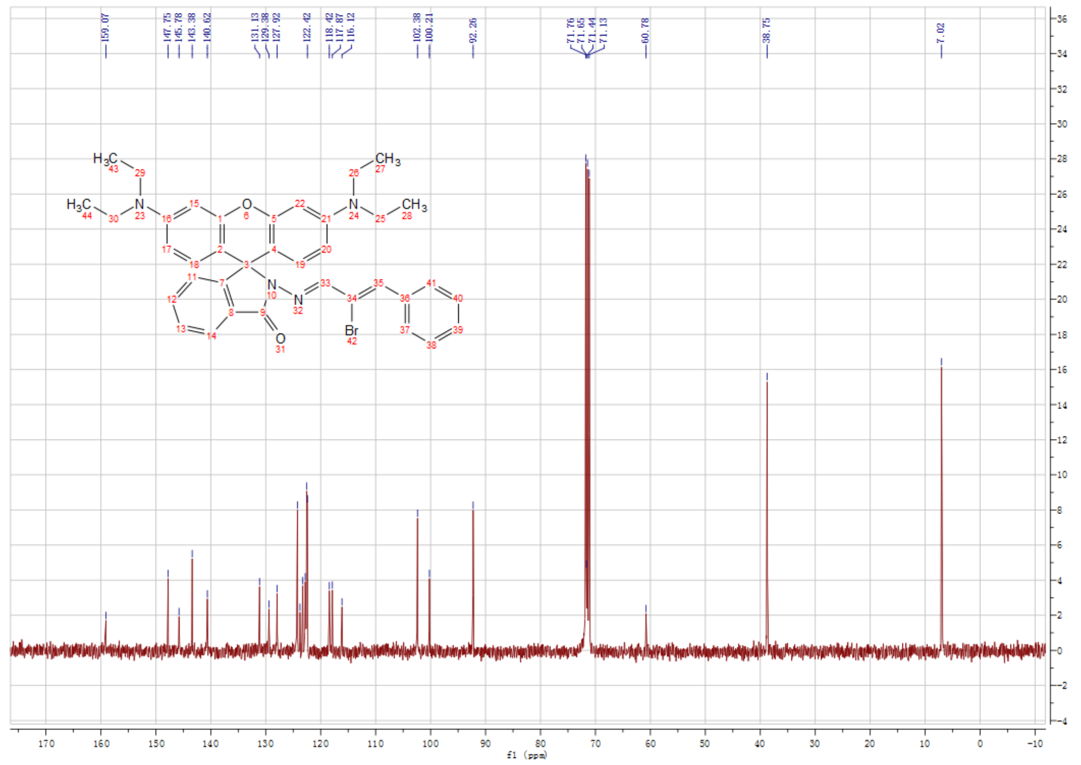

Fig. S36 ^{13}C NMR spectrum of **1d** in $CDCl_3$.

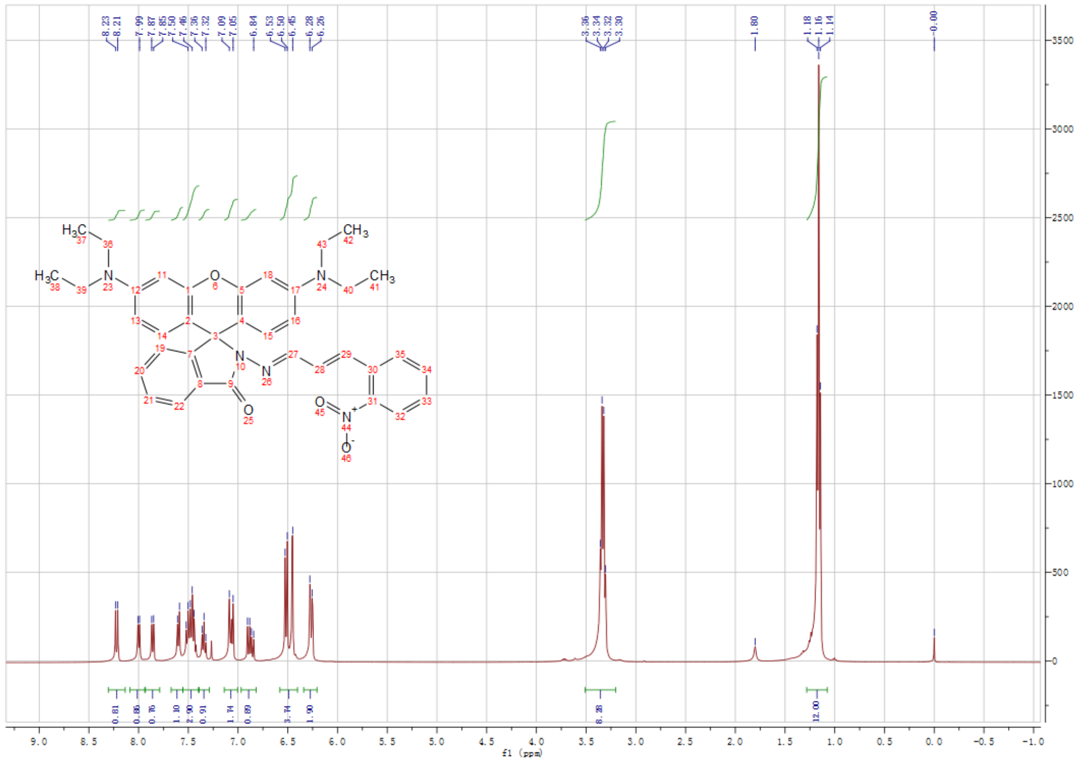

Fig. S37 ^{1}H NMR spectrum of **1e** in $CDCl_3$.

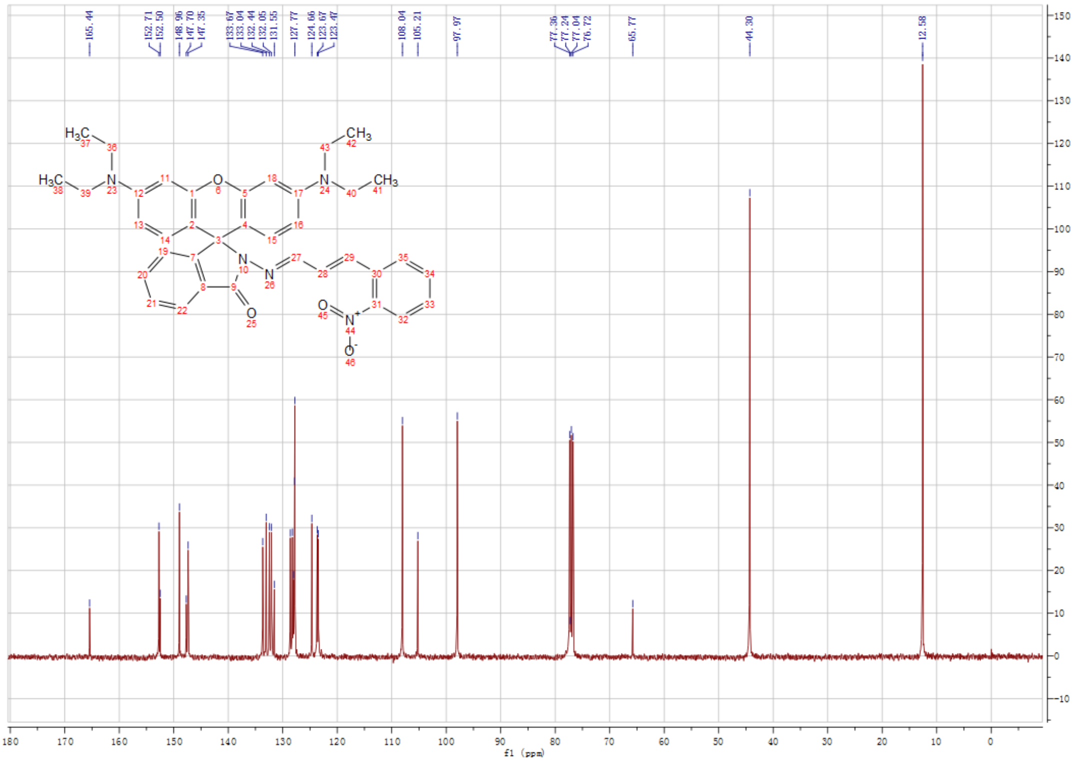

Fig. S38 ^{13}C NMR spectrum of **1e** in $CDCl_3$.

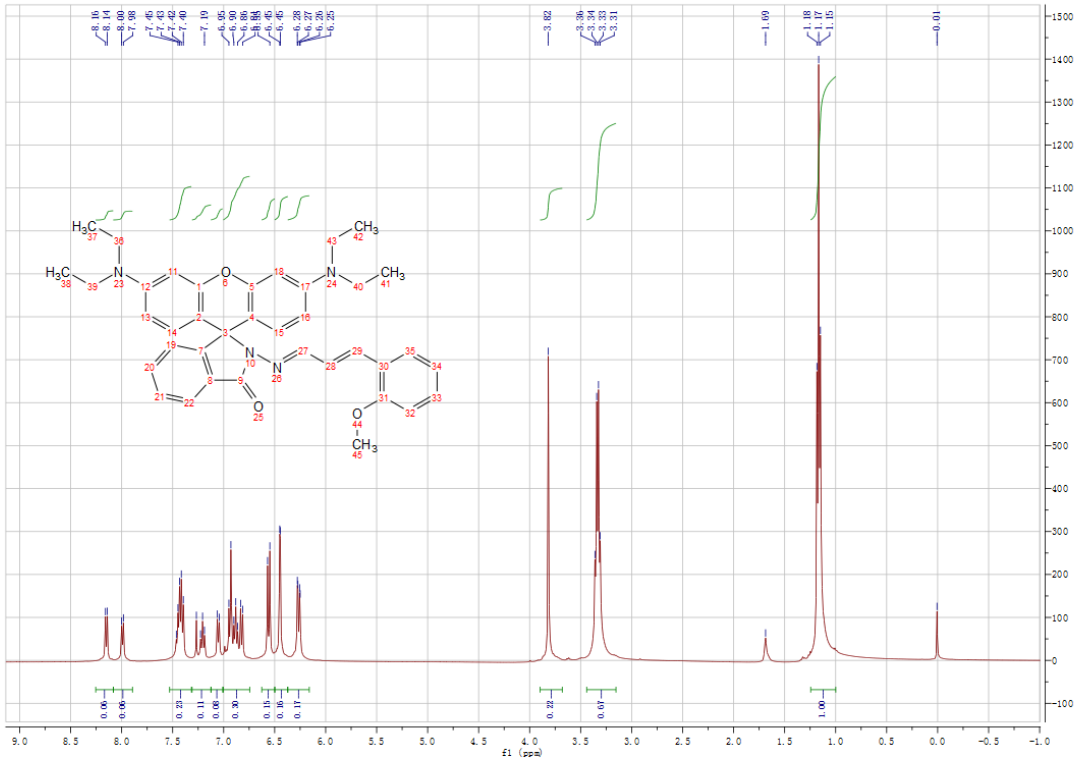

Fig. S39 ^{1}H NMR spectrum of **1f** in $CDCl_3$.

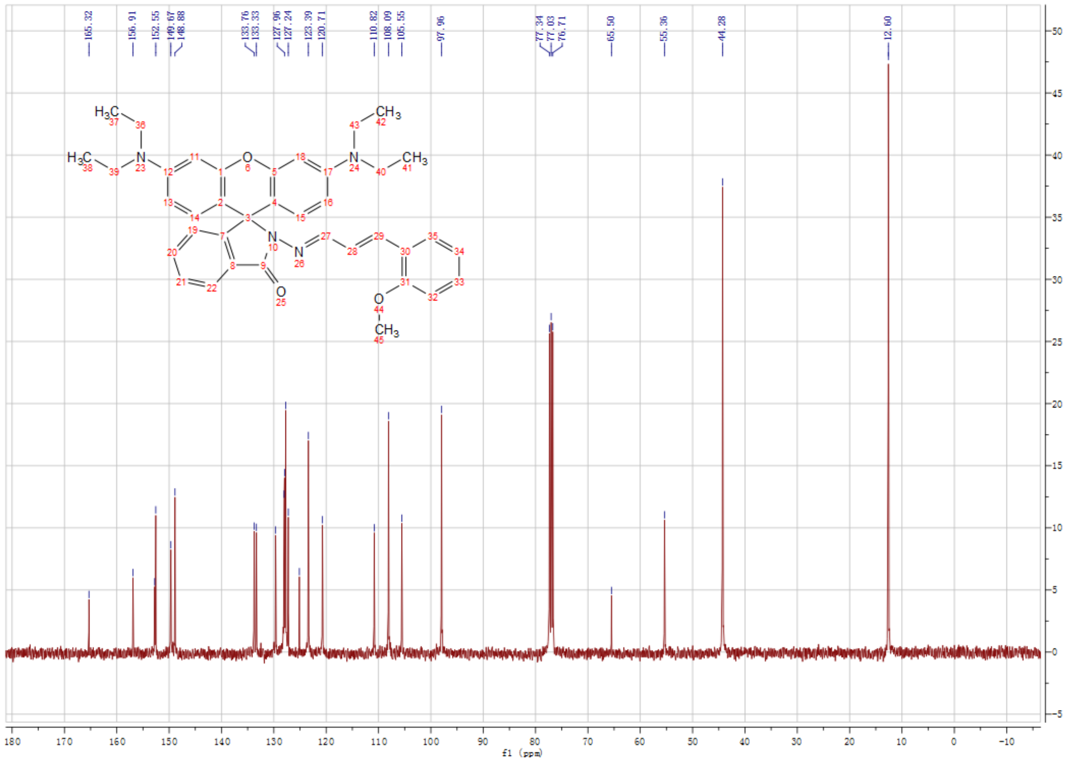

Fig. S40 ^{13}C NMR spectrum of **1f** in $CDCl_3$.

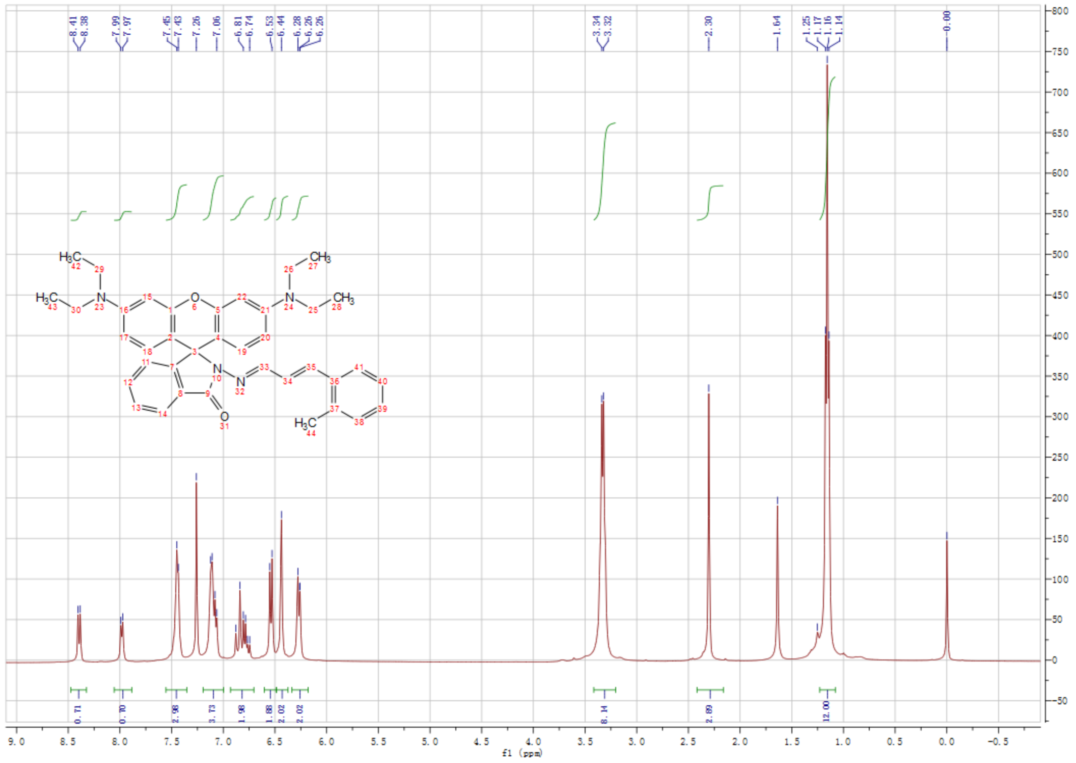

Fig. S41 ^{1}H NMR spectrum of **1g** in $CDCl_3$.

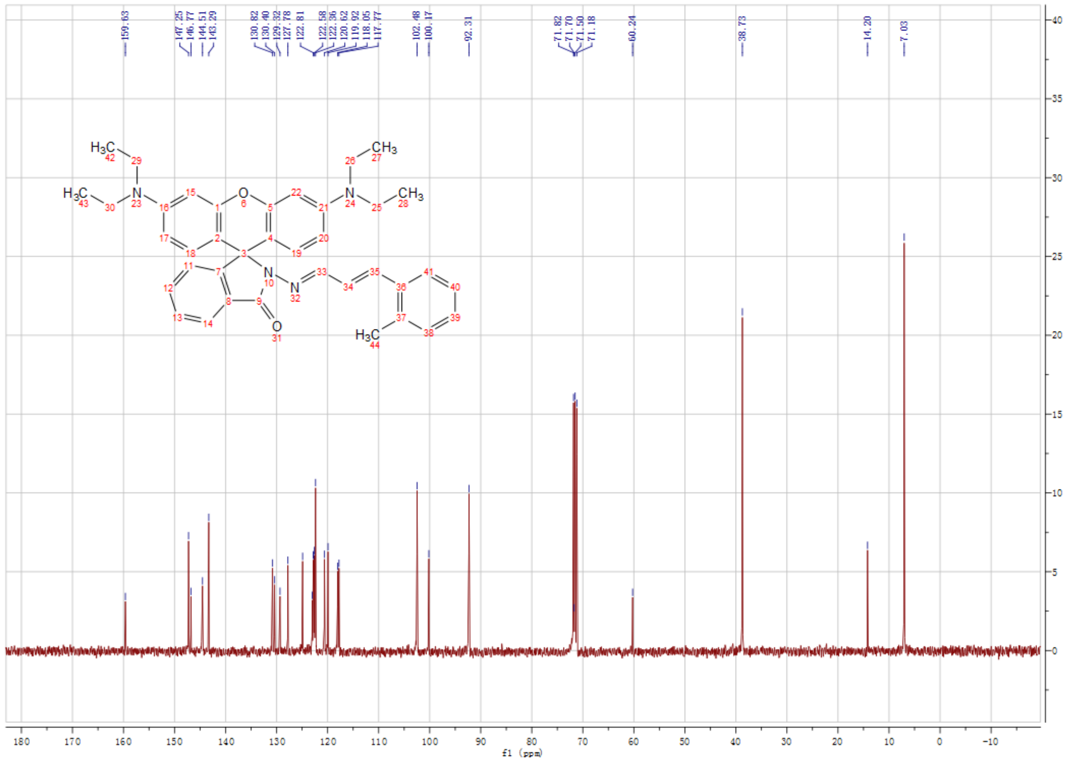

Fig. S42 ^{13}C NMR spectrum of **1g** in $CDCl_3$.

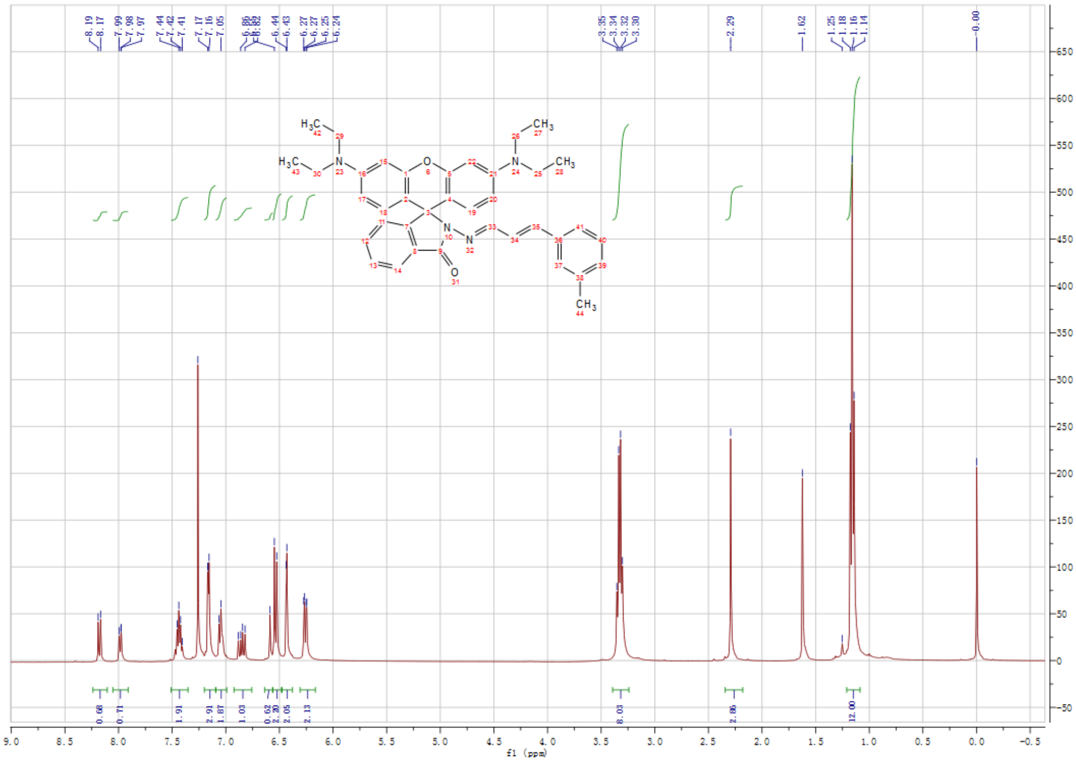

Fig. S43 ^{1}H NMR spectrum of **1h** in $CDCl_3$.

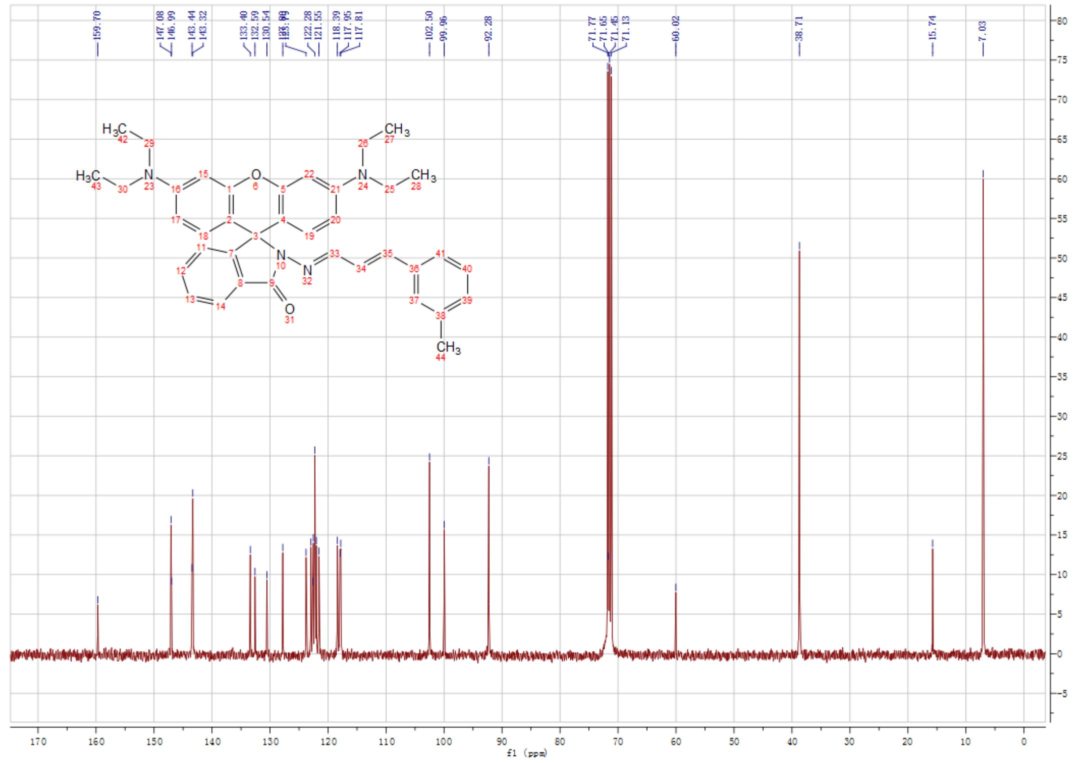

Fig. S44 ^{13}C NMR spectrum of **1h** in $CDCl_3$.

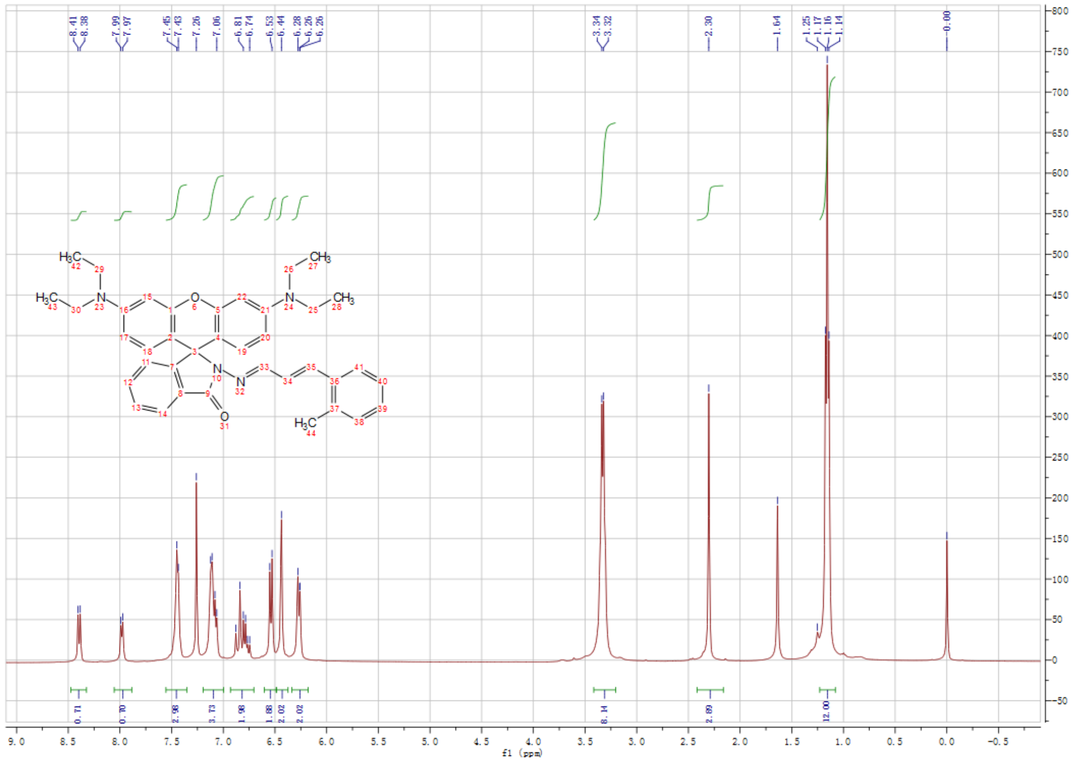

Fig. S45 ^{1}H NMR spectrum of **1i** in $CDCl_3$.

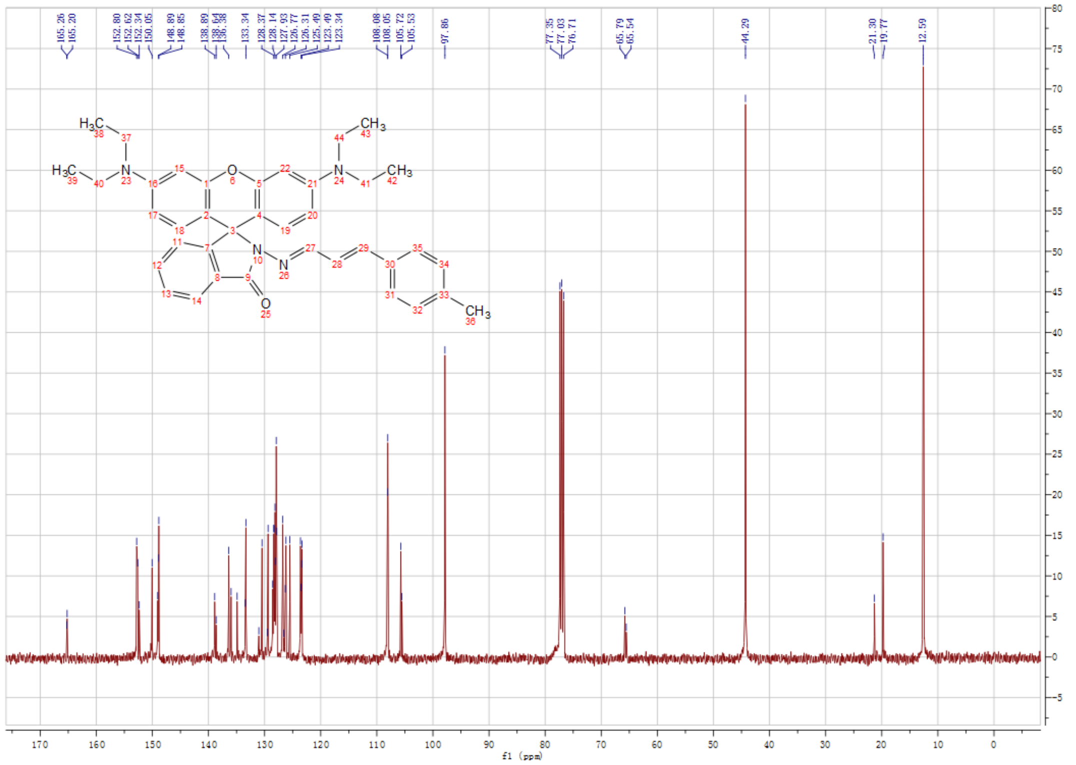

Fig. S46 ^{13}C NMR spectrum of **1i** in $CDCl_3$.

Mass Spectrum List Report

Analysis Info

Analysis Name	C:\Users\lenovo\Desktop\lijianli_yangzheng_1a.d	Acquisition Date	2011/11/28 15:49:01
Method	tune_low 50-500.m	Operator	NWU
Sample Name		Instrument / Ser#	micrOTOF-Q II 10280
Comment			

Acquisition Parameter

Source Type	ESI	Ion Polarity	Positive	Set Nebulizer	0.4 Bar
Focus	Not active	Set Capillary	4500 V	Set Dry Heater	180 °C
Scan Begin	50 m/z	Set End Plate Offset	-500 V	Set Dry Gas	4.0 l/min
Scan End	3000 m/z	Set Collision Cell RF	110.0 Vpp	Set Divert Valve	Source

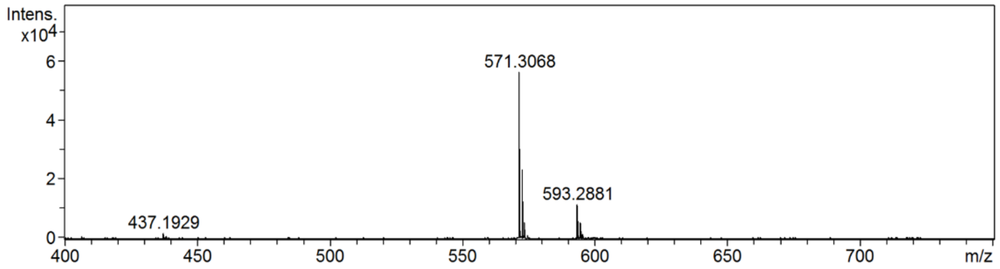

Fig. S47 Mass spectrum of **1a**.

Mass Spectrum List Report

Analysis Info

Analysis Name	C:\Users\lenovo\Desktop\lijianli_yangzheng_1b.d	Acquisition Date	2011/11/28 15:50:37
Method	tune_low 50-500.m	Operator	NWU
Sample Name		Instrument / Ser#	micrOTOF-Q II 10280
Comment			

Acquisition Parameter

Source Type	ESI	Ion Polarity	Positive	Set Nebulizer	0.4 Bar
Focus	Not active	Set Capillary	4500 V	Set Dry Heater	180 °C
Scan Begin	50 m/z	Set End Plate Offset	-500 V	Set Dry Gas	4.0 l/min
Scan End	3000 m/z	Set Collision Cell RF	110.0 Vpp	Set Divert Valve	Source

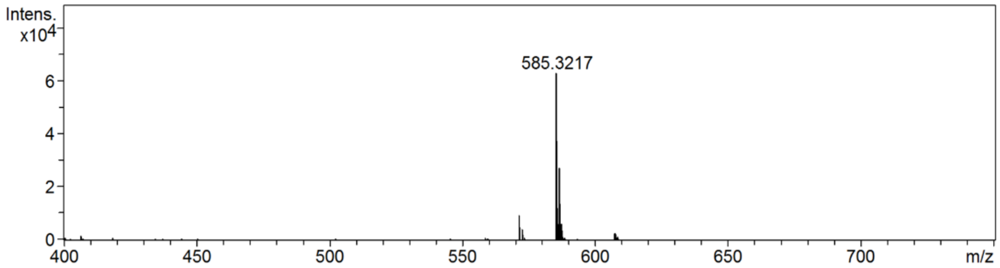

Fig. S48 Mass spectrum of **1b**.

Mass Spectrum List Report

Analysis Info

Analysis Name	C:\Users\lenovo\Desktop\lijianli_yangzheng_1c.d	Acquisition Date	2011/11/28 15:53:53
Method	tune_low 50-500.m	Operator	NWU
Sample Name		Instrument / Ser#	micrOTOF-Q II 10280
Comment			

Acquisition Parameter

Source Type	ESI	Ion Polarity	Positive	Set Nebulizer	0.4 Bar
Focus	Not active	Set Capillary	4500 V	Set Dry Heater	180 °C
Scan Begin	50 m/z	Set End Plate Offset	-500 V	Set Dry Gas	4.0 l/min
Scan End	3000 m/z	Set Collision Cell RF	110.0 Vpp	Set Divert Valve	Source

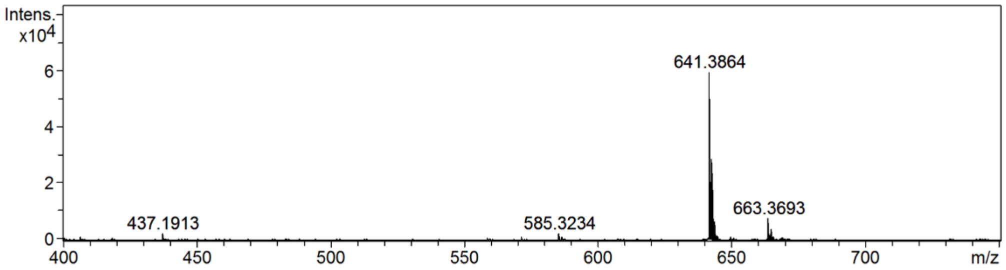

Fig. S49 Mass spectrum of **1c**.

Mass Spectrum List Report

Analysis Info

Analysis Name	C:\Users\Kimi\Desktop\MS-20130524\ljl_21.d	Acquisition Date	2013/5/24 14:42:21
Method	tune_low 50-500.m	Operator	NWU
Sample Name		Instrument / Ser#	micrOTOF-Q II 10280
Comment			

Acquisition Parameter

Source Type	ESI	Ion Polarity	Positive	Set Nebulizer	0.4 Bar
Focus	Not active	Set Capillary	4500 V	Set Dry Heater	180 °C
Scan Begin	50 m/z	Set End Plate Offset	-500 V	Set Dry Gas	4.0 l/min
Scan End	3000 m/z	Set Collision Cell RF	110.0 Vpp	Set Divert Valve	Source

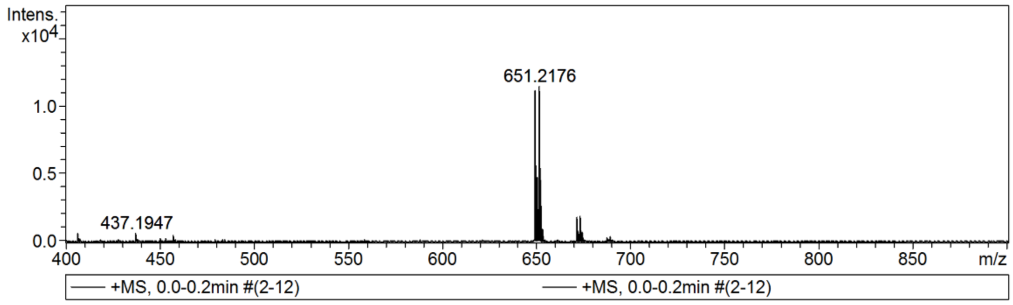

Fig. S50 Mass spectrum of **1d**.

Mass Spectrum List Report

Analysis Info

Analysis Name	C:\Users\Kimi\Desktop\MS-20130524\ljl_22.d	Acquisition Date	2013/5/24 14:45:02
Method	tune_low 50-500.m	Operator	NWU
Sample Name		Instrument / Ser#	micrOTOF-Q II 10280
Comment			

Acquisition Parameter

Source Type	ESI	Ion Polarity	Positive	Set Nebulizer	0.4 Bar
Focus	Not active	Set Capillary	4500 V	Set Dry Heater	180 °C
Scan Begin	50 m/z	Set End Plate Offset	-500 V	Set Dry Gas	4.0 l/min
Scan End	3000 m/z	Set Collision Cell RF	110.0 Vpp	Set Divert Valve	Source

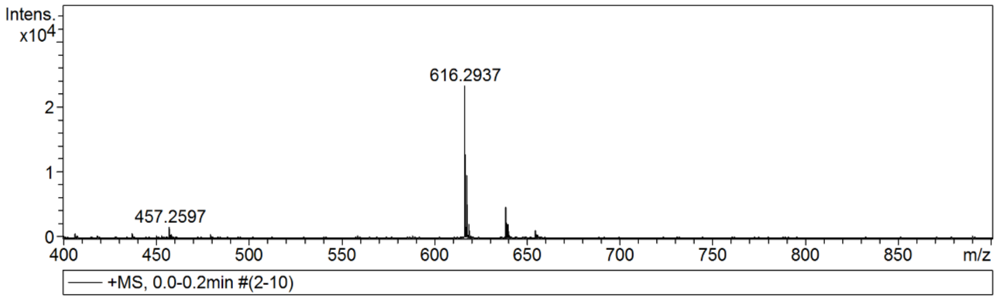

Fig. S51 Mass spectrum of **1e**.

Mass Spectrum List Report

Analysis Info

Analysis Name	C:\Users\Kimi\Desktop\MS-20130524\ljl_12.d	Acquisition Date	2013/5/24 14:15:01
Method	tune_low 50-500.m	Operator	NWU
Sample Name		Instrument / Ser#	micrOTOF-Q II 10280
Comment			

Acquisition Parameter

Source Type	ESI	Ion Polarity	Positive	Set Nebulizer	0.4 Bar
Focus	Not active	Set Capillary	4500 V	Set Dry Heater	180 °C
Scan Begin	50 m/z	Set End Plate Offset	-500 V	Set Dry Gas	4.0 l/min
Scan End	3000 m/z	Set Collision Cell RF	110.0 Vpp	Set Divert Valve	Source

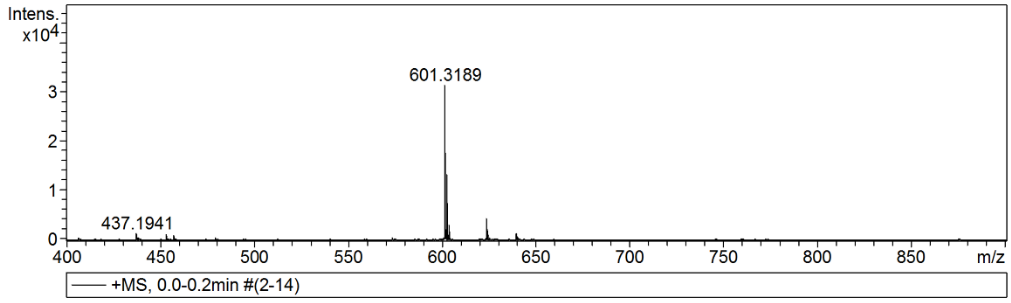

Fig. S52 Mass spectrum of **1f**.

Mass Spectrum List Report

Analysis Info

Analysis Name	C:\Users\Kimi\Desktop\MS-20130524\ljl_17.d	Acquisition Date	2013/5/24 14:31:04
Method	tune_low 50-500.m	Operator	NWU
Sample Name		Instrument / Ser#	micrOTOF-Q II 10280
Comment			

Acquisition Parameter

Source Type	ESI	Ion Polarity	Positive	Set Nebulizer	0.4 Bar
Focus	Not active	Set Capillary	4500 V	Set Dry Heater	180 °C
Scan Begin	50 m/z	Set End Plate Offset	-500 V	Set Dry Gas	4.0 l/min
Scan End	3000 m/z	Set Collision Cell RF	110.0 Vpp	Set Divert Valve	Source

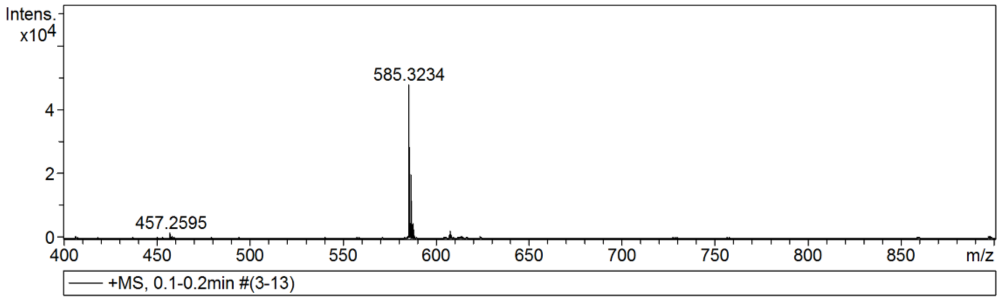

Fig. S53 Mass spectrum of **1g**.

Mass Spectrum List Report

Analysis Info

Analysis Name	C:\Users\Kimi\Desktop\MS-20130524\ljl_24.d	Acquisition Date	2013/5/24 14:50:36
Method	tune_low 50-500.m	Operator	NWU
Sample Name		Instrument / Ser#	micrOTOF-Q II 10280
Comment			

Acquisition Parameter

Source Type	ESI	Ion Polarity	Positive	Set Nebulizer	0.4 Bar
Focus	Not active	Set Capillary	4500 V	Set Dry Heater	180 °C
Scan Begin	50 m/z	Set End Plate Offset	-500 V	Set Dry Gas	4.0 l/min
Scan End	3000 m/z	Set Collision Cell RF	110.0 Vpp	Set Divert Valve	Source

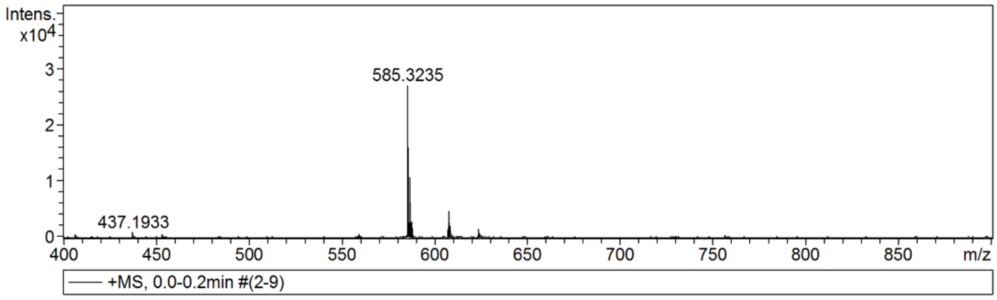

Fig. S54 Mass spectrum of **1h**.

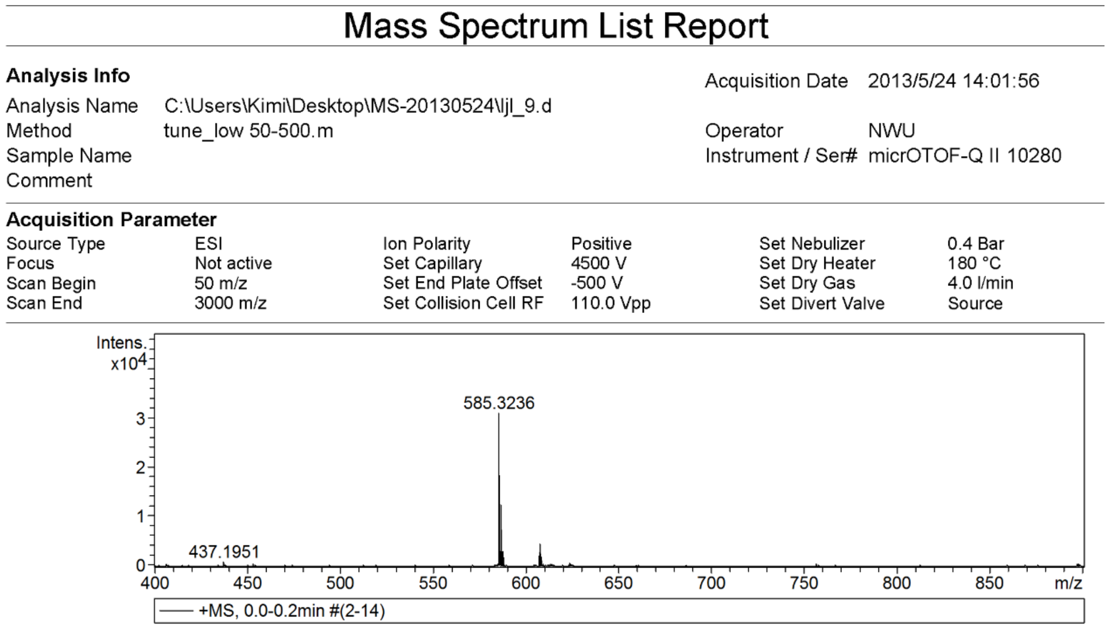

Fig. S55 Mass spectrum of **1i**.

7. Data, spectrum and spectroscopic properties of **1j** for comparison

1j: yellow powder, mp: 125-126 ℃. MS (ESI) m/z = 605.2686 $[M+H]^+$, calc. for $C_{37}H_{38}N_4O_2$ = 604.2605.

^{1}H NMR (400 MHz, $CDCl_3$) δ (ppm) = 8.42 (s, 1H), 7.99 (d, *J* = 6.6 Hz, 1H), 7.70 (d, *J* = 7.3 Hz, 2H), 7.55 - 7.43 (m, 2H), 7.37 - 7.30 (m, 2H), 7.30 (s, 1H), 7.12 (d, *J* = 7.1 Hz, 1H), 6.71 (s, 1H), 6.51 (d, *J* = 8.8 Hz, 2H), 6.44 (d, *J* = 2.5 Hz, 2H), 6.26 (dd, *J* = 8.8, 2.5 Hz, 2H), 3.33 (q, *J* = 7.1 Hz, 8H), 1.16 (t, *J* = 7.0 Hz, 12H).

^{13}C NMR (101 MHz, $CDCl_3$) δ 164.8, 153.2, 151.7, 149.0, 145.0, 134.3, 133.5, 130.0, 129.7, 129.0, 128.8, 128.3, 128.0, 123.9, 123.5, 108.1, 105.7, 97.9, 66.2, 44.4, 12.6.

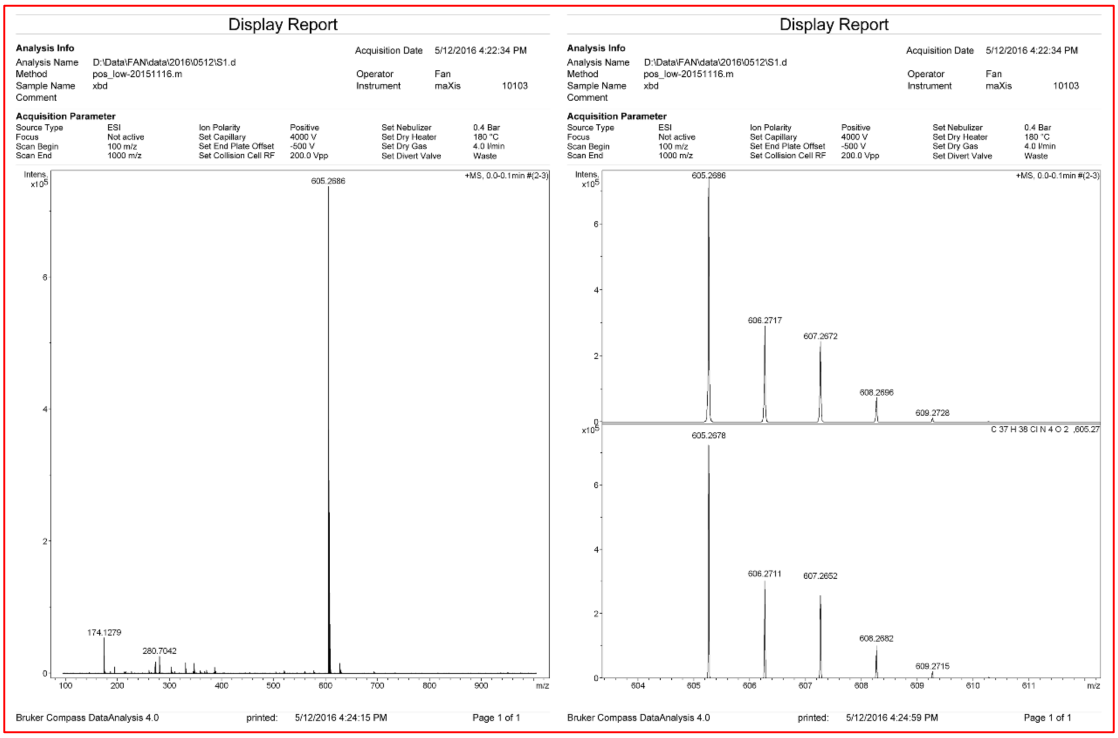

Figure S56 Mass spectrum of **1j**

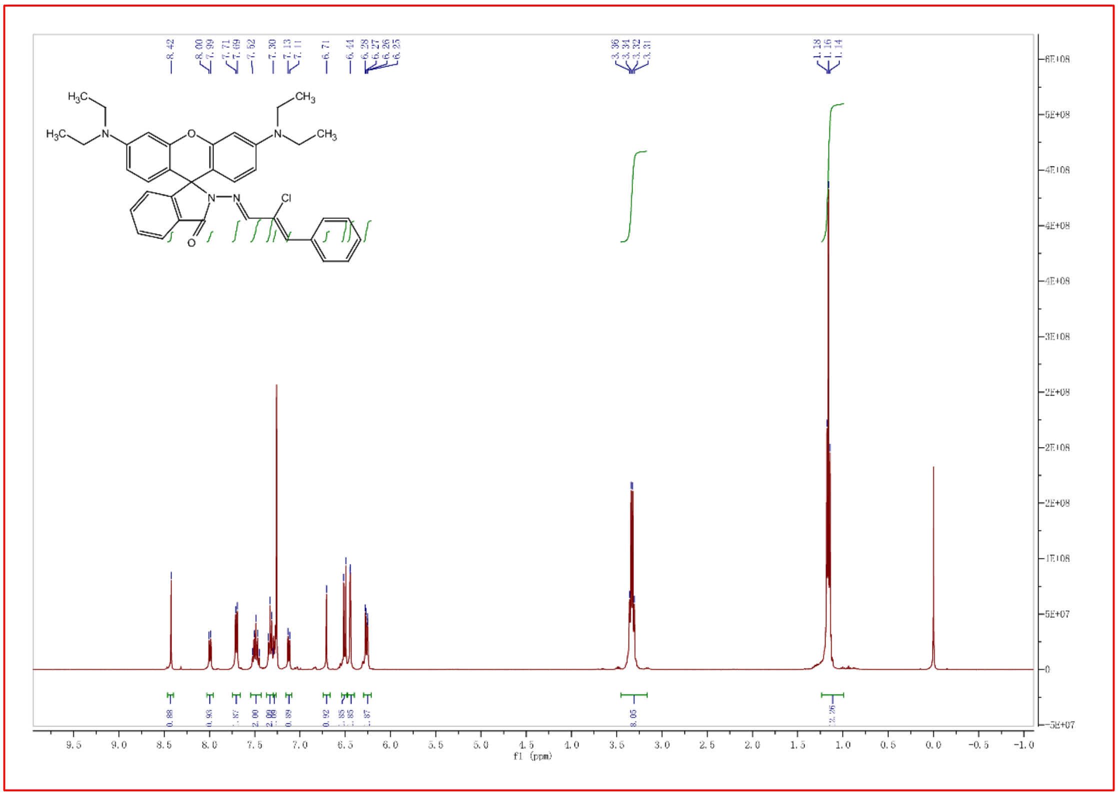

Figure S57 ^{1}H NMR of $C_{37}H_{37}ClN_4O_2$

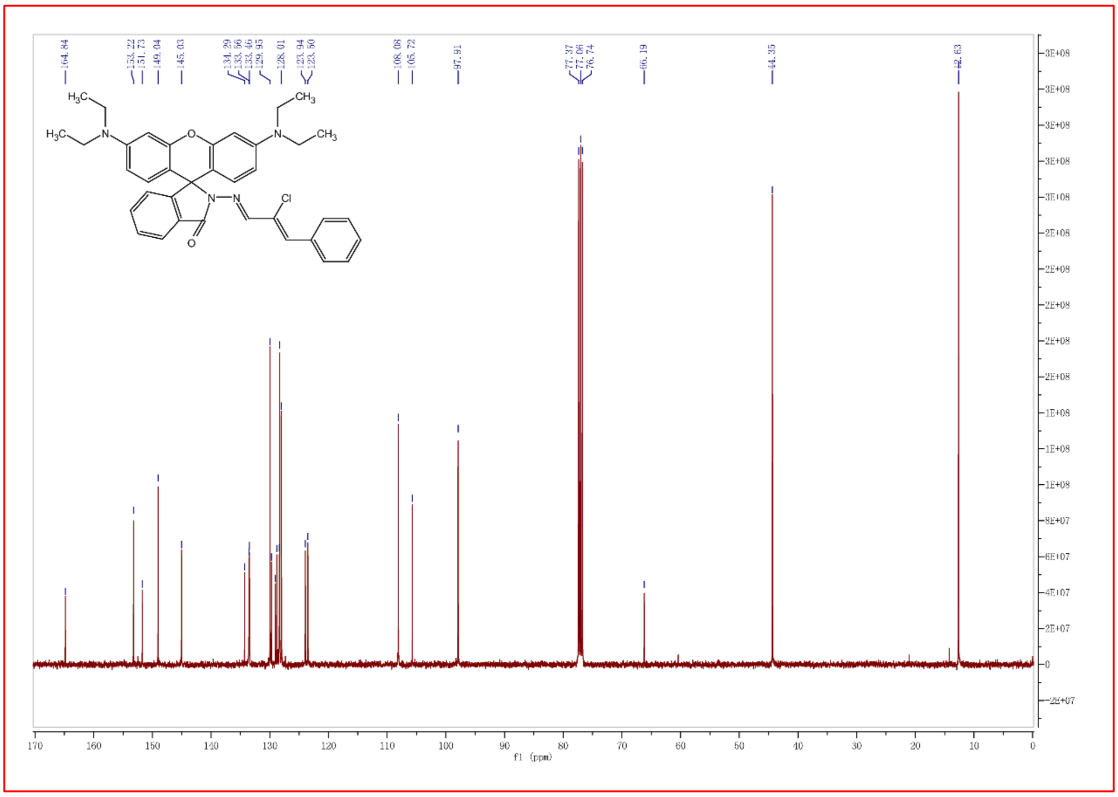

Figure S58 ^{13}C NMR of $C_{37}H_{37}ClN_4O_2$,

The optical properties of probe **1j** is similar to probe **1a-1i** except for the fluorescence intensity and absorption strength. Compared with probe **1d**, **1j** has the weaker fluorescence intensity and higher absorption strength, and the tests are showed in **Figure R8**, all the test conditions and measuring parameters are completely consistent with other probes.

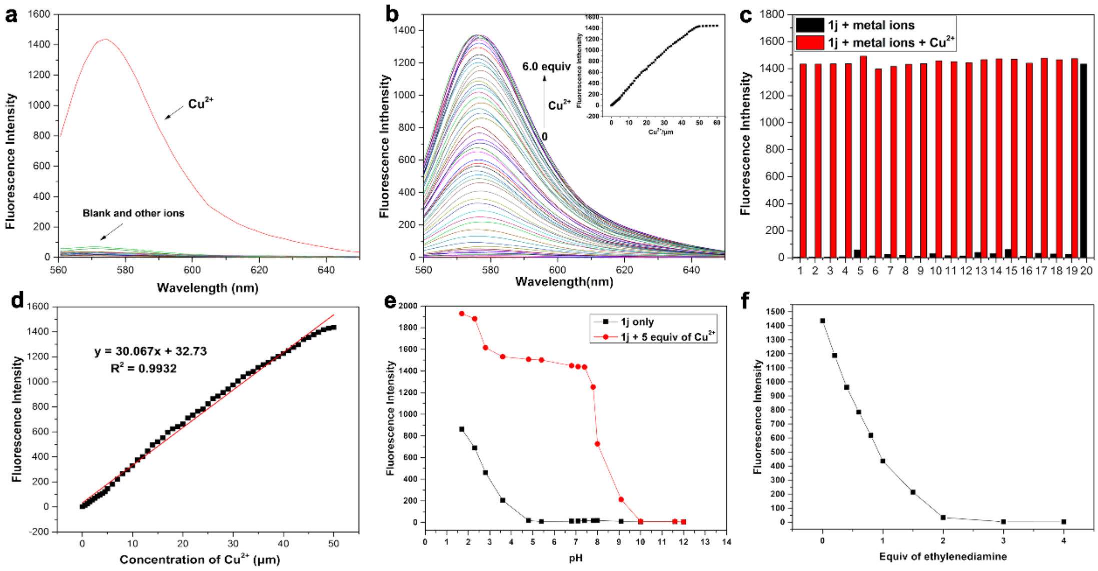

Figure S59 fluoresence properties of probe **1j** (10 μM in ethanol-PBS (5/5, v/v, pH 7.4)), λex = 550 nm, λem max = 576 nm. a: fluorescence spectra of **1j** upon addition of various metal ions (50 μM); b: fluorescence intensity changes of **1j** upon addition of Cu^{2+} (0-50 μM). Inset: Changes in the

emission intensity at 576 nm; c: fluorescence intensity changes of **1j** upon the addition of 50 μM various metal ions in and without the presence of Cu^{2+} (50 μM). 1, blank; 2, Li^{+}; 3, Na^{+}; 4, K^{+}; 5, Ag^{+}; 6, Ba^{2+}; 7, Ca^{2+}; 8, Mg^{2+}; 9, Cd^{2+}; 10, Mn^{2+}; 11, Co^{2+}; 12, Fe^{2+}; 13, Ni^{2+}; 14, Zn^{2+}; 15, Pb^{2+}; 16, Hg^{2+}; 17, Fe^{3+}; 18, Cr^{3+}; 19, Al^{3+}; 20, Cu^{2+}; d: linear relation of fluorescence changes of **1j** with 0 - 50 μM Cu^{2+} at 576 nm; e: fluorescnece intensity of **1j** in the absence and presence of 50 μM Cu^{2+} with different pH conditions; f: fluorescence intensity changes of **1j** upon the addition of ethylenediamine with the presence of Cu^{2+} (50 μM).

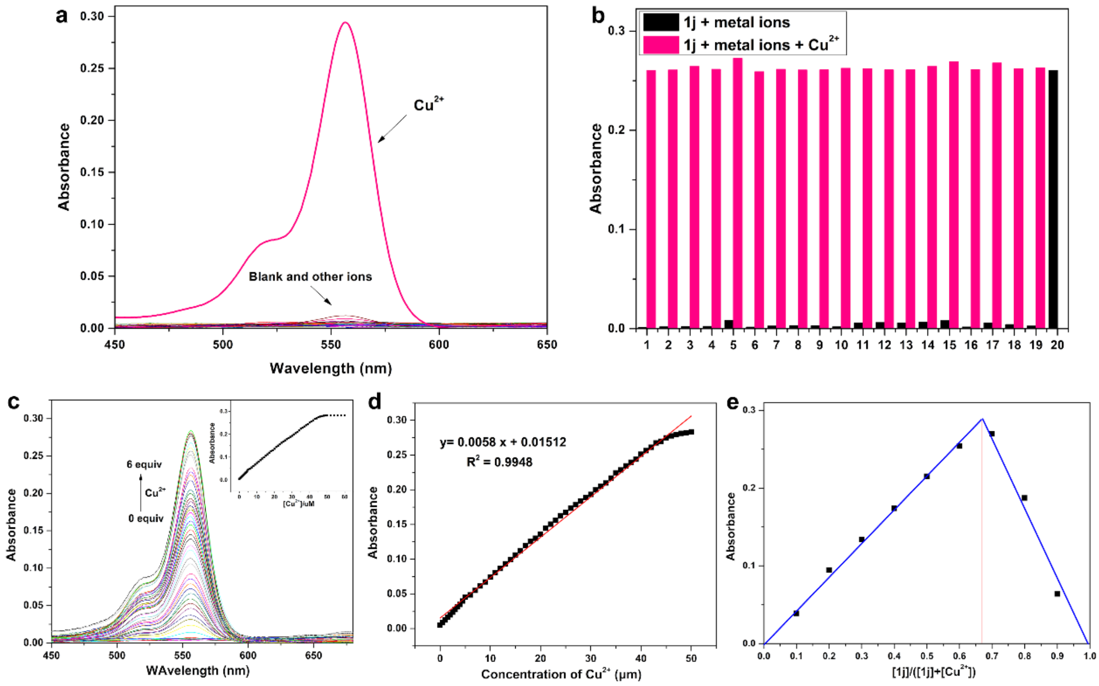

Figure S60 absorption properties of probe **1j** (10 μM in ethanol-PBS (5/5, v/v, pH 7.4)), a: absorption spectra of **1j** upon addition of various metal ions (50 μM); b: absorption strength changes of **1j** upon the addition of 50 μM various metal ions in and without the presence of Cu^{2+} (50 μM) at 556 nm. 1, blank; 2, Li^{+}; 3, Na^{+}; 4, K^{+}; 5, Ag^{+}; 6, Ba^{2+}; 7, Ca^{2+}; 8, Mg^{2+}; 9, Cd^{2+}; 10, Mn^{2+}; 11, Co^{2+}; 12, Fe^{2+}; 13, Ni^{2+}; 14, Zn^{2+}; 15, Pb^{2+}; 16, Hg^{2+}; 17, Fe^{3+}; 18, Cr^{3+}; 19, Al^{3+}; 20, Cu^{2+}; c: absorption changes of **1j** upon addition of Cu^{2+} (0-50 μM). Inset: Changes in the absorption strength at 556 nm; d: linear relation of absorption changes of **1j** with 0 - 50 μM Cu^{2+} at 556 nm; e: job's plot of **1j** and Cu^{2+}, the total concentration of **1j** and Cu^{2+} was 20 μM, the absorbance was measured at 556 nm.

8. Full citation information of Gaussian 09 program

Frisch, M. J.; Trucks, G. W.; Schlegel, H. B.; Scuseria, G. E.; Robb, M. A.; Cheeseman, J. R.;

Scalmani, G.; Barone, V.; Mennucci, B.; Petersson, G. A.; Nakatsuji, H.; Caricato, M.; Li, X.; Hratchian, H. P.; Izmaylov, A. F.; Bloino, J.; Zheng, G.; Sonnenberg, J. L.; Hada, M.; Ehara, M.; Toyota, K.; Fukuda, R.; Hasegawa, J.; Ishida, M.; Nakajima, T.; Honda, Y.; Kitao, O.; Nakai, H.; Vreven, T.; Montgomery Jr., J. A.; Peralta, J. E.; Ogliaro, F.; Bearpark, M. J.; Heyd, J.; Brothers, E. N.; Kudin, K. N.; Staroverov, V. N.; Kobayashi, R.; Normand, J.; Raghavachari, K.; Rendell, A. P.; Burant, J. C.; Iyengar, S. S.; Tomasi, J.; Cossi, M.; Rega, N.; Millam, N. J.; Klene, M.; Knox, J. E.; Cross, J. B.; Bakken, V.; Adamo, C.; Jaramillo, J.; Gomperts, R.; Stratmann, R. E.; Yazyev, O.; Austin, A. J.; Cammi, R.; Pomelli, C.; Ochterski, J. W.; Martin, R. L.; Morokuma, K.; Zakrzewski, V. G.; Voth, G. A.; Salvador, P.; Dannenberg, J. J.; Dapprich, S.; Daniels, A. D.; Farkas, Ö.; Foresman, J. B.; Ortiz, J. V.; Cioslowski, J.; Fox, D. J. *Gaussian 09, Revision A.02*, Gaussian, Inc.: Wallingford, CT, USA, 2009.

References:

1 Bourson, J., Pouget, J. & Valeur, B. Ion-responsive fluorescent compounds. 4. Effect of cation binding on the photophysical properties of a coumarin linked to monoaza- and diaza-crown ethers. *The Journal of Physical Chemistry* **97**, 4552-4557, doi:10.1021/j100119a050 (1993).

2 Shao, N. *et al.* Copper Ion-Selective Fluorescent Sensor Based on the Inner Filter Effect Using a Spiropyran Derivative. *Analytical Chemistry* **77**, 7294-7303, doi:10.1021/ac051010r (2005).

3 Zhao, Y. *et al.* Highly Sensitive and Selective Colorimetric and Off−On Fluorescent Chemosensor for Cu^{2+} in Aqueous Solution and Living Cells. *Analytical Chemistry* **81**, 7022-7030, doi:10.1021/ac901127n (2009).

4 Sar, D., Bag, R., Yashmeen, A., Bag, S. S. & Punniyamurthy, T. Synthesis of Functionalized Pyrazoles via Vanadium-Catalyzed C–N Dehydrogenative Cross-Coupling and Fluorescence Switch-On Sensing of BSA Protein. *Organic Letters* **17**, 5308-5311, doi:10.1021/acs.orglett.5b02669 (2015).

5 Tsukamoto, K., Shimabukuro, S., Mabuchi, M. & Maeda, H. A Naphthalimide-Based Cd^{2+} Fluorescent Probe with Carbamoylmethyl Groups Working as Chelators and PET-Promoters under Neutral Conditions. *Chemistry – A European Journal*, n/a-n/a, doi:10.1002/chem.201600556 (2016).

6 Zhou, H. *et al.* Highly Selective Fluorescent Recognition of Sulfate in Water by Two Rigid Tetrakisimidazolium Macrocycles with Peripheral Chains. *Journal of the American Chemical Society* **135**, 14908-14911, doi:10.1021/ja406638b (2013).

7 Wu, D., Huang, W., Duan, C., Lin, Z. & Meng, Q. Highly Sensitive Fluorescent Probe for Selective Detection of Hg^{2+} in DMF Aqueous Media. *Inorganic Chemistry* **46**, 1538-1540, doi:10.1021/ic062274e (2007).

8 Huang, W. *et al.* Recognition Preference of Rhodamine-Thiospirolactams for Mercury(II) in Aqueous Solution. *Inorganic Chemistry* **48**, 5061-5072, doi:10.1021/ic8015657 (2009).

9 Huang, H., Shi, S., Zheng, X. & Yao, T. Sensitive detection for coralyne and mercury ions based on homo-A/T DNA by exonuclease signal amplification. *Biosensors and Bioelectronics* **71**, 439-444, doi:10.1016/j.bios.2015.04.076 (2015).
